# Mucosal Vaccination: A Promising Alternative Against Flaviviruses

**DOI:** 10.3389/fcimb.2022.887729

**Published:** 2022-06-15

**Authors:** Rosendo Luria-Pérez, Luis A. Sánchez-Vargas, Paola Muñoz-López, Gabriela Mellado-Sánchez

**Affiliations:** ^1^ Hospital Infantil de México Federico Gómez, Unidad de Investigación en Enfermedades Hemato-Oncológicas, Ciudad de México, Mexico; ^2^ Department of Cell and Molecular Biology, Institute for Immunology and Informatics, University of Rhode Island, Providence, RI, United States; ^3^ Posgrado en Biomedicina y Biotecnología Molecular, Escuela Nacional de Ciencias Biológicas, Instituto Politécnico Nacional, Ciudad de México, Mexico; ^4^ Unidad de Desarrollo e Investigación en Bioterapéuticos (UDIBI), Escuela Nacional de Ciencias Biológicas, Instituto Politécnico Nacional, Ciudad de México, Mexico; ^5^ Laboratorio Nacional para Servicios Especializados de Investigación, Desarrollo e Innovación (I+D+i) para Farmoquímicos y Biotecnológicos, LANSEIDI-FarBiotec-CONACyT, Ciudad de México, Mexico

**Keywords:** mucosae, vaccine, flavivirus, non-replicating vectors, replicating vectors

## Abstract

The Flaviviridae are a family of positive-sense, single-stranded RNA enveloped viruses, and their members belong to a single genus, Flavivirus. Flaviviruses are found in mosquitoes and ticks; they are etiological agents of: dengue fever, Japanese encephalitis, West Nile virus infection, Zika virus infection, tick-borne encephalitis, and yellow fever, among others. Only a few flavivirus vaccines have been licensed for use in humans: yellow fever, dengue fever, Japanese encephalitis, tick-borne encephalitis, and Kyasanur forest disease. However, improvement is necessary in vaccination strategies and in understanding of the immunological mechanisms involved either in the infection or after vaccination. This is especially important in dengue, due to the immunological complexity of its four serotypes, cross-reactive responses, antibody-dependent enhancement, and immunological interference. In this context, mucosal vaccines represent a promising alternative against flaviviruses. Mucosal vaccination has several advantages, as inducing long-term protective immunity in both mucosal and parenteral tissues. It constitutes a friendly route of antigen administration because it is needle-free and allows for a variety of antigen delivery systems. This has promoted the development of several ways to stimulate immunity through the direct administration of antigens (e.g., inactivated virus, attenuated virus, subunits, and DNA), non-replicating vectors (e.g., nanoparticles, liposomes, bacterial ghosts, and defective-replication viral vectors), and replicating vectors (e.g., *Salmonella enterica*, *Lactococcus lactis*, *Saccharomyces cerevisiae*, and viral vectors). Because of these characteristics, mucosal vaccination has been explored for immunoprophylaxis against pathogens that enter the host through mucosae or parenteral areas. It is suitable against flaviviruses because this type of immunization can stimulate the parenteral responses required after bites from flavivirus-infected insects. This review focuses on the advantages of mucosal vaccine candidates against the most relevant flaviviruses in either humans or animals, providing supporting data on the feasibility of this administration route for future clinical trials.

## 1. Introduction

### 1.1 Flavivirus Overview

The genus *Flavivirus* of the family Flaviviridae consists of over 70 small (~50-nm diameter), positive-sense, single-stranded RNA enveloped viruses of which at least 33 are known to infect humans.

The genus includes Dengue virus (DENV), Japanese Encephalitis virus (JEV), West Nile virus (WNV), Zika virus (ZIKV), Tick-borne Encephalitis virus (TBEV), Yellow Fever virus (YFV), Kyasanur Forest disease (KFD), Duck Tembusu virus (DTMUV), and Louping ill virus (LIV), which cause significant diseases worldwide (Lindenbach and Rice, 2003; Medin and Rothman, 2017). The clinical conditions present during human flavivirus infection can include hemorrhagic syndromes, neurological disorders, congenital malformations, and fetal death, although most symptomatic flavivirus infections result in self-limiting flu-like febrile illness (Medin and Rothman, 2017; Pierson and Diamond, 2020). Most of the known flaviviruses are transmitted to vertebrates by infected hematophagous mosquitoes or ticks (Blitvich and Firth, 2017).

Flaviviruses share a common genomic organization, polyprotein processing, and particle structure, although they differ in the natural host, cellular tropism, and transmission cycles (Kuno et al., 1998; Billoir et al., 2000; Gaunt et al., 2001; Kuno, 2007; Gould and Solomon, 2008; Blitvich and Firth, 2015). The flavivirus genome (approximately 11 Kb) codifies a single polyprotein cleaved by both viral and host proteases into ten viral proteins. Three structural proteins, a capsid (C), a pre-membrane (PrM), and an envelope (E), mediate virus attachment and entry; while seven nonstructural (NS) proteins (NS1, NS2A, NS2B, NS3, NS4A, NS4B, and NS5) are involved in viral genome replication and the assembly of new viral particles (Lindenbach and Rice, 2003). Most flaviviruses exist as a single serotype, except for the DENV group, composed of four closely related serotypes known as DENV1-4. Their amino acid sequences share a similarity of 65–70% within the E protein (Green and Rothman, 2006).

There are no currently approved therapeutic antivirals for the treatment of human flavivirus infection (Holbrook, 2017). Vector control is effective for limiting flavivirus diseases, yet there are substantial limitations for its use (Shaw and Catteruccia, 2019). Vaccination is the most useful approach to reducing the burden of human disease caused by flavivirus. Nonetheless, only five flavivirus vaccines (DENV, YFV, JEV, TBEV, and KFDV) are licensed for human use (Gould and Solomon, 2008; Ishikawa et al., 2014; Collins and Metz, 2017; Shah et al., 2018).

### 1.2 DENV

Dengue, transmitted to humans by infected *Aedes* mosquitoes, remains the most important arthropod-borne viral disease worldwide (Bhatt et al., 2013; Brady and Hay, 2020). Infections by DENV are a significant cause of morbidity, mortality, and economic impact in more than 100 tropical and subtropical countries. It is estimated that 2.5 billion people are at risk of infection with limited prevention options (Bhatt et al., 2013), while approximately 390 million infections and 21,000 deaths occurs annually, mostly among children (Thomas and Endy, 2011; Bhatt et al., 2013). The existence and cocirculation of multiple DENV serotypes allow the same individual to experience more than one infection in a lifetime. The spectrum of the disease ranges from subclinical infection and self-limited dengue fever to life-threatening dengue hemorrhagic fever and dengue shock syndrome (Guzman and Kouri, 2003). A recent classification according to the severity defines dengue infection as dengue without warning signs, dengue with warning signs, and severe dengue (Hadinegoro, 2012). The infection with any DENV serotype elicits lifelong homotypic immunity and temporary cross immunity against the other serotypes; however, secondary infections increase the probability of severe dengue disease (Sabin, 1952; Guzman et al., 2013).

### 1.3 JEV

Over 4 billion people in Asia are vulnerable to infection by JEV, the major cause of viral encephalitis (Yun and Lee, 2014). Infected *Culex* mosquitoes are the principal JEV vector. The virus is a single serotype with five genotypes (Mackenzie et al., 2004), while the infection may present as asymptomatic or symptomatic, with meningitis, encephalitis, and flaccid paralysis, affecting mostly infants and children (Solomon and Vaughn, 2002). There are approximately 68,000 cases annually, with a 20–30% mortality rate, and 30–50% of the survivors suffer long-term neurological and psychiatric sequelae (Moore, 2021).

### 1.4 WNV

First isolated from a febrile woman in the West Nile district of Uganda in 1937, WNV is currently the most widespread arbovirus. It is found in many areas of Africa, West Asia, the Middle East, Europe, Australia, and North America (May et al., 2011). Transmission to humans occurs mainly by infected *Culex* mosquitoes and person-to-person through organ transplantation or blood and blood product transfusion (Mackenzie et al., 2004). While most people infected with WNV have mild or no symptoms, some present West Nile fever (20%) and less than 1% develop severe neurological disease. Severe disease is rare in infants and children, and severe encephalitis and death occur most commonly in the elderly (Sejvar, 2014).

### 1.5 ZIKV

ZIKV, the virus most closely related to DENV, is a re-emerging arthropod-borne flavivirus that circulates in the same geographic areas as DENV (Ngono and Shresta, 2018). It is commonly transmitted to humans by infected *Aedes* mosquitoes, although mother to fetus during pregnancy and sexual contact transmissions have been reported. ZIKV was first isolated from a rhesus monkey in Uganda in 1947 and humans in 1952, yet it gained global attention recently after outbreaks reported in the Yap Islands State, Federated States of Micronesia (2007), French Polynesia (2013), and Brazil (2015) (Duffy et al., 2009; Lowe et al., 2018; Musso et al., 2018). Most of the people infected with ZIKV experience an asymptomatic or self-limiting disease; still, the virus may cause neurological disorders and congenital malformations (Lowe et al., 2018).

### 1.6 TBEV

Endemic in Europe and Asia, TBEV produces more than 10,000 infections every year. The virus is transmitted to humans through the saliva of infected *Ixodes* ticks. Three closely related groups of TBEV (European, Siberian, and Far Eastern) cause infections that can range from asymptomatic and mild conditions to severe neurological disease and death (Mansfield et al., 2009).

### 1.7 YFV

Yellow Fever (YF) remains endemic in more than 45 countries located in Africa, Central, and South America, with an estimate of 200,000 severe cases and 60,000 deaths every year despite an effective vaccine (Chen and Wilson, 2020). Spread by infected *Aedes* mosquitoes, YFV mainly affects humans and nonhuman primates. The clinical presentation of YF varies from asymptomatic to febrile illness that may result in hepatitis, renal failure, hemorrhage, and shock (Gardner and Ryman, 2010).

### 1.8 KFDV

Kyasanur Forest disease (KFD) is a viral hemorrhagic disease caused by KFDV. The virus was first identified and isolated from a sick monkey from the Kyasanur Forest in the Shimoga district, Karnataka, India, in 1957 (Work, 1958). The virus is transmitted to humans through the bite of infected *Haemaphysalis spinigera* ticks and affects 400–500 humans per year. It causes severe hemorrhagic fever with neurological manifestations, with a fatality rate of 3–10% (Shah et al., 2018).

### 1.9 DTMUV

An emerging pathogenic flavivirus named duck Tembusu virus (DTMUV), transmitted by mosquitoes, caused a major outbreak in Chinese duck farms in 2010 (Su et al., 2011). Although DTMUV does not cause disease in humans, antibodies against DTMUV were detected in serum samples of duck industry workers. The infection results in severe egg-drop production and neurological disorders with high morbidity and mortality (Tang et al., 2013).

### 1.10 LIV

A flavivirus found almost exclusively in Great Britain, LIV primarily affects sheep. It is transmitted to humans by *Ixodes ricinus* ticks, causing an asymptomatic disease that rarely results in a severe neurological condition. To date, only one fatal case has been reported in humans (Jeffries et al., 2014).

Better vaccines must be developed due to the epidemiological relevance and the biological differences between flaviviruses (e.g., complexity and pathological outcomes), along with the insufficient vector control to manage the diseases they cause. In this work, we first review the immunological aspects to consider before developing vaccines; then, we present a summary of the licensed and relevant flavivirus vaccines which are or have been in clinical trials. Finally, we review the advantages of mucosal immunization and the current state of the art of the different flavivirus vaccine platforms using a mucosal route. This work aims to provide the elements of judgment to evaluate the feasibility of mucosal vaccination against human or animal flaviviruses.

## 2. Immune Response to Flavivirus Infections

The human immune responses, both innate and adaptive, induced by flaviviruses are mostly protective against disease and essential to virus clearance. Most of the infections result in subclinical or self-limiting flu-like febrile illness.

The innate immune system provides the first line of defense against flaviviruses. Since most flaviviruses infect humans *via* an insect bite in the skin, dendritic cells (DC) located in the skin are usually the first immune cells to interact with these viruses (Diamond, 2003). Pattern recognition receptors as toll-like receptors (TLR3 and TLR7) and retinoic acid-inducible gene I (RIG-I)-like receptors, such as melanoma differentiation-associated gene 5 (MDA5), RIG-I, and cyclic GMP-AMP synthase (cGAS), play key roles in sensing viral RNA, which is a central mechanism of the innate immune response. Effective RNA recognition is crucial for triggering the appropriate antiviral response, which includes the synthesis and secretion of type-I and II interferons (IFNs) that promote an antiviral state and contribute to flavivirus clearance (Chang et al., 2006; Fredericksen et al., 2008; Nasirudeen et al., 2011; Chazal et al., 2018).

On the other hand, the innate immune response helps to establish adaptive immunity, which contributes to the resolution of the infection and protects from reinfection with the same flavivirus. Conversely, the pre-existing immunity to a specific flavivirus (which is cross-reactive among flaviviruses) can influence the clinical outcome in a subsequent heterologous flavivirus infection with possible immunopathological activity (Slon Campos et al., 2018; Sanchez Vargas et al., 2020). This phenomenon is described as follows.

### 2.1 Flavivirus Cross-Reactive Antibody Responses

Antibodies are a critical part of the adaptive immune response against infecting flaviviruses. The primary mechanism of protection against flavivirus is through the neutralization of their particles by antibodies that limit the infection spread (Rey et al., 2018; Slon Campos et al., 2018). In addition, anti-flavivirus antibodies can contribute to viral clearance by Fc-mediated mechanisms, such as antibody-dependent cellular cytotoxicity (ADCC), antibody-dependent cellular phagocytosis (ADCP), and antibody-dependent complement deposition (ADCD) (Kurane et al., 1984; Laoprasopwattana et al., 2007; Sanchez Vargas et al., 2021).

The antibodies are predominantly directed to the E, PrM, and NS1 proteins (Rey et al., 2018). The E protein is the main target of neutralizing antibodies in flavivirus infections. These antibodies inhibit the attachment to the cellular receptors, entry into the host cells, or the viral replication (Pierson et al., 2008; Dowd and Pierson, 2011). Anti-PrM antibodies are highly cross-reactive but poorly neutralizing, whereas anti-NS1 antibodies elicit host protection *via* ADCC and ADCD (Dejnirattisai et al., 2010; Slon Campos et al., 2018; Reyes-Sandoval and Ludert, 2019; Sanchez Vargas et al., 2021).

The antibody response to flavivirus is type-specific and cross-reactive (Rathore and St John, 2020). An example of cross protection between flaviviruses occurs with DENV and ZIKV, which share a high degree of homology. Antibodies from a previous DENV infection protect against a symptomatic ZIKV infection (Montecillo-Aguado et al., 2019; Rodriguez-Barraquer et al., 2019). However, antibodies from a prior ZIKV infection enhance DENV infections *via* antibody-dependent enhancement (ADE) (Dejnirattisai et al., 2016; Stettler et al., 2016; Katzelnick et al., 2020a; Katzelnick et al., 2020b). The ADE hypothesis suggests that cross-reacting and poorly neutralizing antibodies or neutralizing antibodies at sub-neutralizing concentrations from a previous flavivirus infection potentially exacerbate the disease with the same flavivirus or a related one (OhAinle et al., 2011; Dejnirattisai et al., 2016; Rey et al., 2018).

### 2.2 Flavivirus Cross-Reactive T cell Responses

Both CD4^+^ and CD8^+^ T cells contribute to the eradication of virally infected cells during a flavivirus infection (Slon Campos et al., 2018). Subsets of CD4^+^ T cells are known to promote the elimination of the virus by cooperating with CD8^+^ T cells. They produce antiviral cytokines and help B cells to produce antibodies. It was recently found that some CD4^+^ T cell subtypes could lyse infected cells, preventing the spread of infection (Weiskopf et al., 2015; Saron et al., 2018; Sanchez-Vargas and Mathew, 2019). Flavivirus-specific CD8^+^ T cells mainly respond by killing infected target cells through different mechanisms as perforin and granzymes, Fas–Fas ligand, and TNF-related apoptosis-inducing ligand (TRAIL) interactions. Additionally, anti-viral cytokine production, mostly IFN-γ and tumor necrosis factor α (TNF-α), has been documented (Hatch et al., 2011; Weiskopf et al., 2013).

The responses of CD4^+^ and CD8^+^ T cells against peptides in structural and non-structural flavivirus proteins tend to be highly cross-reactive against flaviviruses (Rivino et al., 2013; Schwaiger et al., 2014; Turtle et al., 2016; Koblischke et al., 2017; Reynolds et al., 2018). Closely related to each other, DENV and ZIKV share approximately 55% of their amino acid sequence identity (Ngono and Shresta, 2018). There are differences in immunodominance across flaviviruses; for example, ZIKV structural proteins (E, PrM, and C) are major targets of CD4^+^ and CD8^+^ T cell responses, whereas DENV T cell epitopes are found primarily in nonstructural proteins (Grifoni et al., 2017). The specificity of T cells could be altered in sequential flavivirus infections (Grifoni et al., 2017; Saron et al., 2018). DENV and ZIKA virus-specific T cell responses are highly cross-reactive against each other (Wen et al., 2017).

In addition to protection, cross-reactive memory T cells can have pathological consequences during reactivation, depending on the context. Cross-reactive memory T cells from a prior flavivirus infection dominate the response to the subsequent infecting heterologous flavivirus. The response tends to be less effective, with suboptimal degranulation and reduced cytolytic activity, but high in inflammatory cytokine production leading to a cytokine storm (Klenerman and Zinkernagel, 1998; Mongkolsapaya et al., 2003; Mangada and Rothman, 2005; Dong et al., 2007; Reynolds et al., 2018). As a result, T cells can promote immunopathology, increasing the risk for severe disease in a second flavivirus exposure (Mangada et al., 2002; Mongkolsapaya et al., 2003; Sanchez-Vargas et al., 2021).

## 3. Flavivirus Vaccines, Licensed or in Clinical Trials

According to the World Health Organization, manufacturers must develop vaccines that “are effective in preventing or reducing the severity of infectious disease; provide durable, long-term protection against the disease; achieve immunity with a minimal number of doses; provide the maximum number of antigens that confer the broadest protection against infection; cause no or mild adverse events; are stable at extremes of storage conditions over a prolonged period of time; are available for general use through mass production; are affordable to populations at risk for infectious disease.” (World Health Organization, 2013)

**Figure 1:**
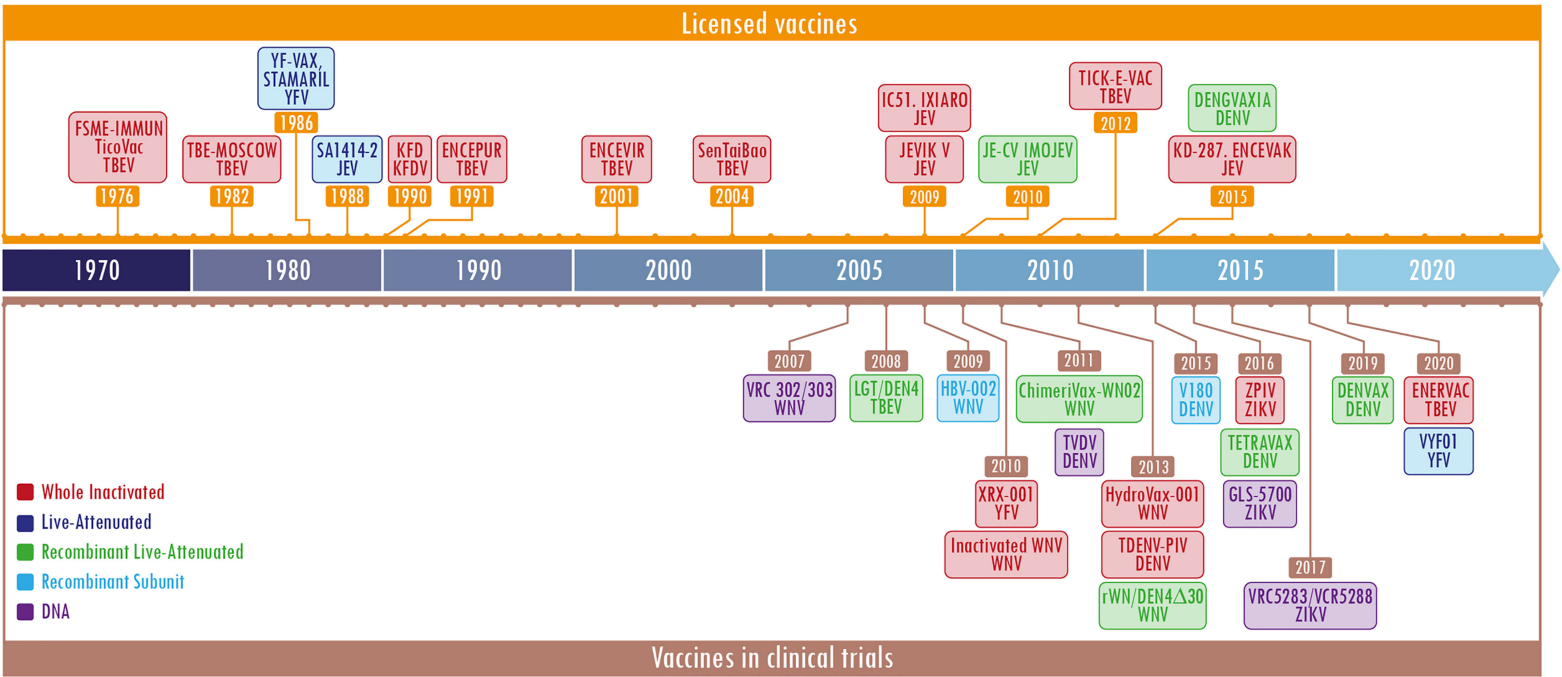
Timeline of licensed flavivirus vaccines and most representative candidate vaccines in clinical trials. The figure is divided into two sections, the upper one (in orange) shows the licensed vaccines and the lower one (in brown) depicts the vaccines in clinical trials. The different platforms employed for the developments are shown in specific colors per label. Each label contains a first or second line with the name of the licensed vaccine or the development, and the last one represents the virus against which the vaccine was designed. The color code is located in the lower section, the left side. The year shown is the licensing date or beginning of the clinical trial. The most representative vaccines under clinical trials were obtained at: https://clinicaltrials.gov/. The keywords used were flavivirus and vaccines.

Currently, flavivirus infections have a significant impact on public health. In the absence of specific and effective antiviral treatments, vaccination and the control of vectors remain critical tools when controlling and preventing flavivirus infections. However, the development of vaccines against these agents faces important challenges as the pre-existing immunity to flaviviruses in endemic regions could provide cross-protection or drastically increase heterologous flavivirus infections (Rey et al., 2018). Several platforms have been tested to generate suitable flaviviruses vaccine candidates (Fischer et al., 2020). According to the type of agent used to activate the immune system, they are classified in inactivated, live attenuated, subunit, and recent platforms (World Health Organization, 2013; Pollard and Bijker, 2021). The dates of licensing and initiation of clinical trials are shown in the timelines in **Figure 1** and **Supplementary Table 1**.

According to the Center for Disease Dynamics, Economics & Policy, seven major emerging and re-emerging infectious disease outbreaks of the past two decades have been caused by ZIKV (https://cddep.org/tool/major-emerging-and-re-emerging-infectious-disease-outbreaks-2002-2020/). This highlights the need for stronger incentives for preparedness and the creation of a global insurance fund to alleviate the economic losses caused by infectious diseases in general. This virus is part of the priority diseases published by WHO that must be addressed in public health contexts (https://www.who.int/activities/prioritizing-diseases-for-research-and-development-in-emergency-contexts). Meanwhile, the outbreaks and endemicity of DENV generate losses of billions of dollars. Therefore, one of the most relevant strategies to control flavivirus diseases is immunoprophylaxis. Vaccinologists must seek to improve licensed vaccines and search for alternatives to parenteral routes of immunization. In the meantime, these infectious diseases will remain a threat to public health and the global economy.

Overall, the currently licensed vaccines against flaviviruses have achieved different levels of efficacy. One of the best vaccines ever developed so far is against YFV, the YF17D attenuated vaccine, which is effective and safe; due to these characteristics, it has been used as a backbone for other vaccines (Guirakhoo et al., 1999). Conversely, Dengvaxia, the licensed vaccine against DENV has rendered different protective responses (around 30-70%) depending on previous immune status, age, and genotypic variation within each serotype that individuals are exposed to (Tully and Griffiths, 2021). Therefore, further improvements to DENV vaccines or against WNV and ZIKV, which do not have licensed vaccines, are mandatories.

Accordingly, vaccines against flavivirus are still under research for better approaches that guarantee a protective immune response, fewer side effects, and a low cost for all populations. In this context, mucosal vaccination emerges as a novel approach that should be considered in order to overcome these issues.

## 4. Advantages of Mucosal Vaccines

Most pathogenic agents penetrate the host through the mucous membranes, composed of an epithelial layer that provides protection, defense, and homeostasis with the external environment. The mucous membranes transport macromolecules and perform secretory and barrier functions, preventing the colonization of pathogenic microorganisms and others. It covers the body’s internal surfaces and constitutes a physical barrier against pathogen invasion. (Fujkuyama et al., 2012).

**Figure 2:**
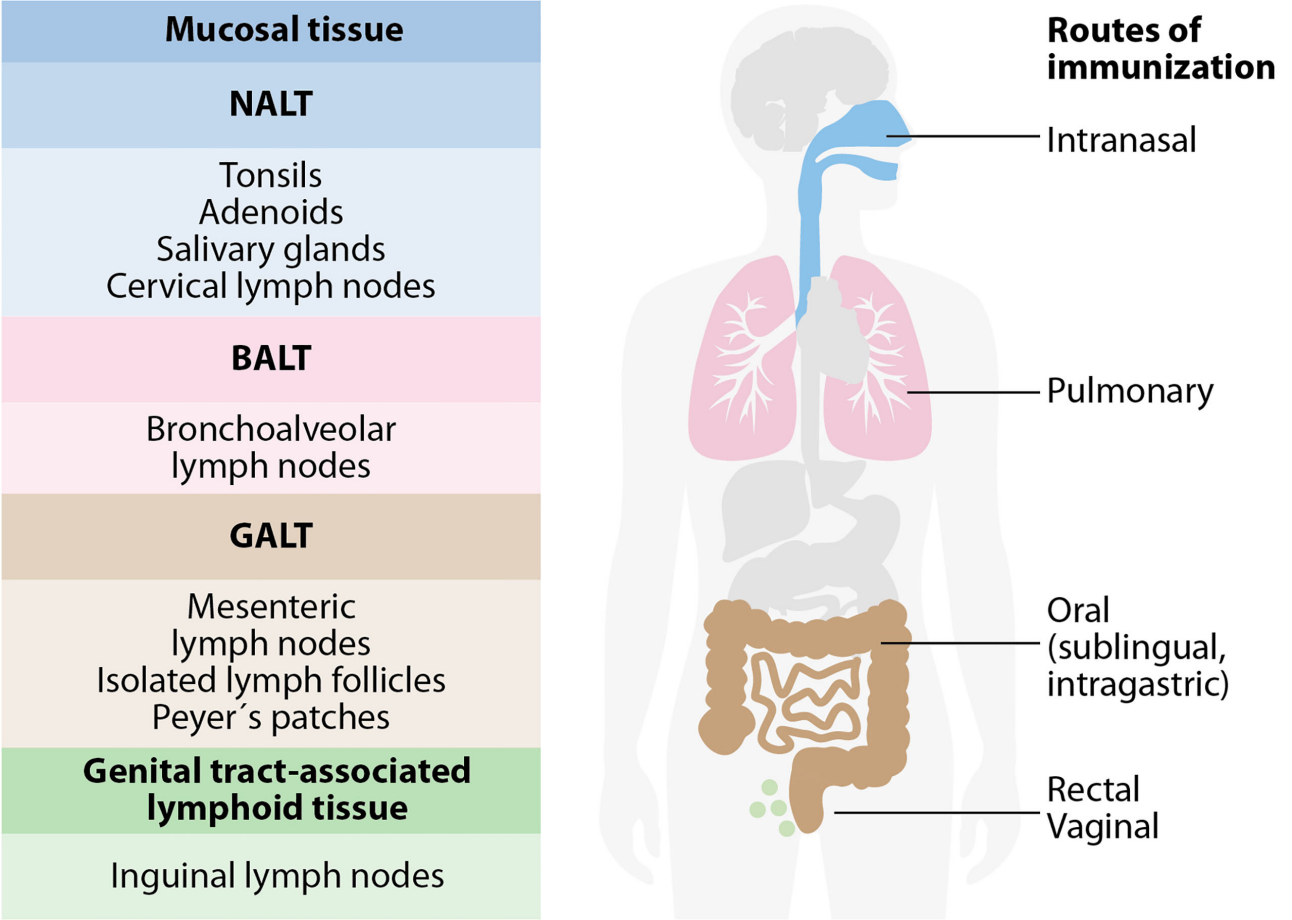
The mucosa-associated lymphoid tissue (MALT). MALT has subcompartments: nasopharynx-associated lymphoid tissue (NALT), bronchus-associated lymphoid tissue (BALT), gut-associated lymphoid tissue (GALT), and genital-associated lymphoid tissue (GENALT). The compartmentalization of mucosal immune responses constrains the selection of vaccine administration routes. Intranasal vaccination is preferred for targeting the respiratory system, while oral and sublingual vaccinations are effective for gut immunity. Rectal immunization allows immunity in the colon, rectum, and the urinary tract to a certain extent. Intravaginal vaccination is the most effective for antibody and T cell immunity in the urinary tract.

The mucosa-associated lymphoid tissue (MALT) is the leading site of induction where immune responses start. This tissue is independent of the systemic immune system and includes all mucus-lined surfaces of the body, associated follicles in the large intestine, Peyer’s patches (PPs), appendix, tonsils, lacrimal glands, salivary glands, conjunctiva, and the lactating sinus. The antigen monitoring takes place on the mucosal surface; then, the priming of B and T cells occurs (**Figure 2**).

**Figure 3:**
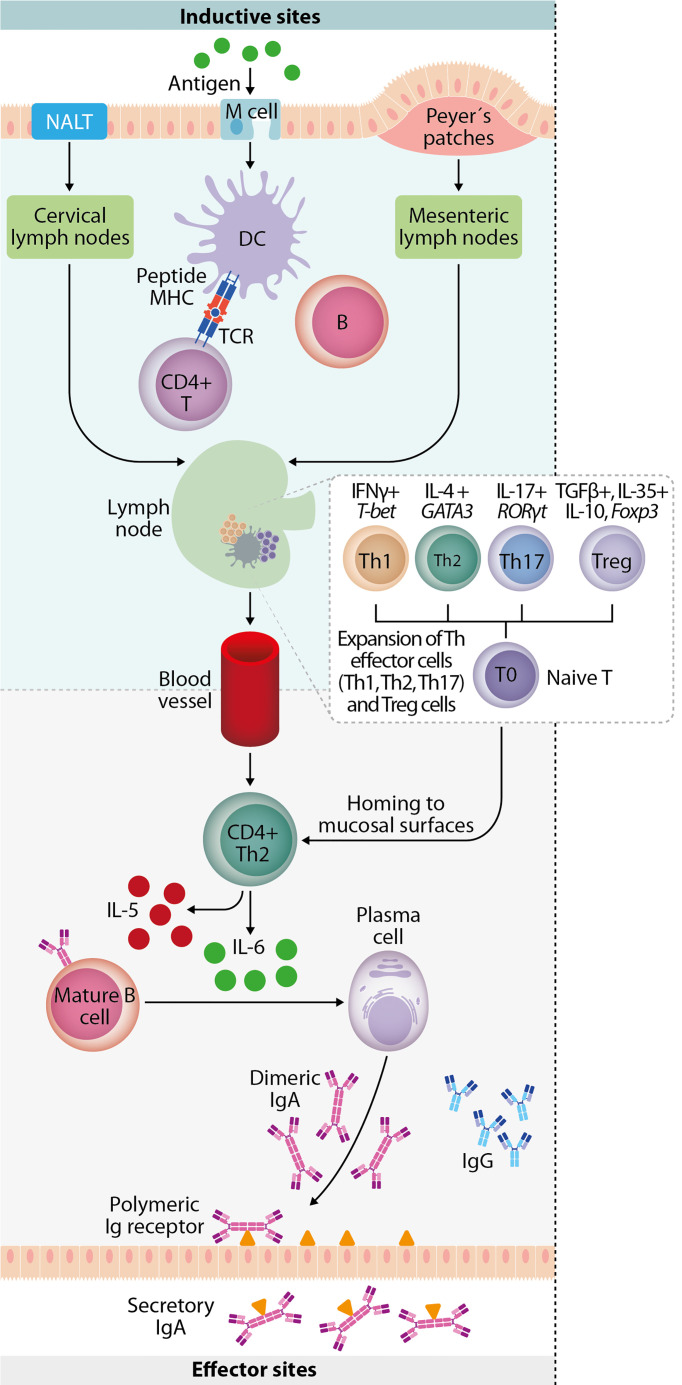
Mucosal immune response. Mucosal immunity plays a crucial role against invading pathogens on the epithelial cell surface, involving a complex network of innate and adaptive immune components. Luminal antigens are transported to the nasopharynx-associated lymphoid tissue (NALT) and gut-associated lymphoid tissue (GALT) through M cells in the epithelium overlying NALT and Peyer’s patches. Mucosal dendritic cells are key to initiating adaptive immune responses by migrating to the draining lymph node and mediating the expansion of antigen-specific naive T-cells into T helper subsets. They involve upregulation of transcription factors (T-bet, GATA-3, RORγt, and Foxp3) and lineage-defining cytokines (IFN-γ, IL-4, IL-17, TGF-β, IL-35, and IL-10). IgA+ B cells and plasmablasts then differentiate into IgA-producing plasma cells in the presence of cytokines (IL-5 and IL-6) produced by T-helper 2 (Th2) cells. They subsequently produce dimeric or polymeric forms of IgA. Finally, antigen-specific CD4^+^ T cells and IgA+ B cells migrate to effector sites (such as nasal passage and intestinal lamina propria) through the thoracic duct and blood circulation.

In addition, MALT is covered by a follicle-associated epithelium consisting of a subset of epithelial cells that are differentiated into microfolds, columnar epithelial cells, and lymphoid cells, which play a central role in initiating mucosal immune responses. Microfold cells (M cells) take up antigen from the intestinal and nasal mucosa lumen and transport it to underlying antigen-presenting cells (APCs), including DC, B lymphocytes, and macrophages. T cells receive antigenic activation and costimulation by APCs that support their clonal expansion and the cytokine cues that dictate their differentiation and homing to peripheral tissues. B cells perform several immunological functions, such as producing antibodies as sIgA, functioning as APCs, and secreting cytokines. B cells can present antigens in the lamina propria (LP) to effector T cells (Holmgren and Czerkinsky, 2005; Lycke, 2012; Li et al., 2020). Moreover, mucosal vaccination can trigger systemic immunoglobulin G (IgG) responses against the antigen. Antigen uptake can induce systemic IgG, while the activated mucosal DC can migrate to the lymph nodes and spleen. The processed antigen is presented to naïve T cells and triggers adaptive immunity. Systemic IgG is also induced when a portion of the B cells are activated in the mucosa and express the peripheral homing receptors α4β1-integrin and leukocyte (L)-selectin, allowing them to migrate to the regional lymph nodes. Humoral immunity and cellular mediated responses are coordinated by CD4^+^ and CD8^+^ T cells, essential against intracellular pathogens, like viruses, as shown in **Figure 3** (Kunkel and Butcher, 2003). As a consequence, mucosal vaccines can activate both arms of the adaptive immune system (De Magistris, 2006). The immunization of one mucosal site results in the secretion of the same specific IgA antibodies into other distal mucosal sites, a phenomenon known as the common mucosal immune system (Kunkel and Butcher, 2003).

Mucosal vaccination aims to prevent the initial colonization and infection by pathogens while eliciting a strong immune response at mucosal sites. It leads to a systemic immune response that includes antibody production and immune cell-mediated responses (Lycke, 2012; Wang et al., 2015).

Mucosal vaccines provide advantages like low reactivity, reduced costs, simple application, and non-invasiveness. Since they require no specially trained health personnel, equipment, or needles. This last characteristic prevents transmissible blood infections due to needle re-use and needle stick injury (Levine, 2003). Mucosal vaccination is ideal for mass application and generates no biohazardous waste, unlike parenteral vaccines (Correia-Pinto et al., 2013; Davitt and Lavelle, 2015).

Although mucosal vaccines came to the spotlight in the 21st century, only a few have been registered. Given the challenges for their development, approved mucosal vaccines constitute a small group that includes influenza A and B viruses, H1N1 influenza virus, poliovirus, rotavirus, cholera, and *Salmonella* Typhi (Harakuni et al., 2009; Zakay-Rones, 2010; Carter and Curran, 2011; Heinonen et al., 2011; Kulkarni et al., 2013; Orenstein, 2015; Kirkwood et al., 2019; Pezzoli, 2020; Yeh et al., 2020). These pathogens enter the host through mucosae and most of the vaccination platforms against them are based on live attenuated microorganisms, highlighting that the best strategy is to emulate the route of natural infection.

Mucosal vaccines can be administered through different routes and induce varied immune responses. The oral and intranasal (IN) routes offer a more practical administration, and they stimulate broad and disseminated antigen-specific mucosal and systemic immune responses. However, the nature of the antigen and its targeted mucosal tissue also affect the efficacy of the vaccines. The IN route can emulate the natural infection of pathogens and elicit specific mucosal and systemic immune responses with relatively low doses of antigen. These routes also avoid first-pass metabolism and reduces the risk of anaphylactic shock. Licensed vaccines have proven that oral immunization is a feasible strategy since these vaccines can induce strong and broad responses. These responses include mucosal secretory IgA (sIgA), neutralizing serum IgG, memory T cells and other synergistic effectors of the immune response (Li et al., 2020). In this context, oral and IN approaches have been evaluated in mucosal vaccines against flavivirus with promising results.

**Figure 4:**
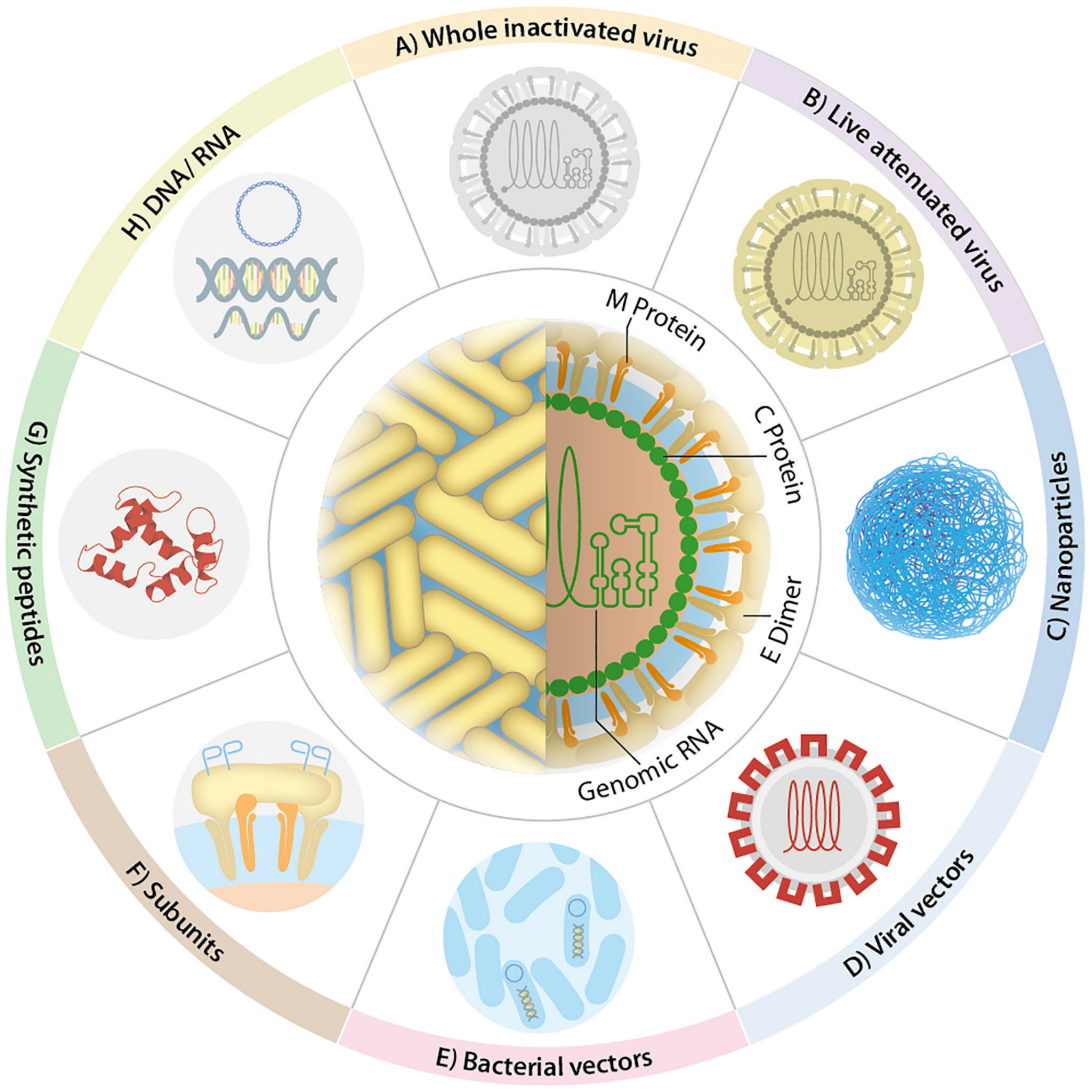
Flavivirus vaccine design. The most representative vaccines against flaviviruses use: **(A)** whole inactive virus, **(B)** live-attenuated virus, **(C)** nanoparticles, **(D)** viral vectors, **(E)** bacterial vectors, **(F)** subunits, **(G)** synthetic peptides, and **(H)** nucleic acids. Other strategies have been included in some of the depicted ones, e.g., liposomes **(C)**, virus-like particles **(D)**, bacterial ghosts **(E)**, fusion proteins **(F)**.

The development of mucosal vaccines should consider oral tolerance and a more significant amount of antigen to induce a potent immune response. Experience from licensed mucosal vaccines has shown that orally or nasally administered vaccines could be supplemented with either naturally-occurring or synthetic adjuvants to overcome those issues (Davitt and Lavelle, 2015; Wang et al., 2015). Moreover, the use of live attenuated strains of bacterial or viral carriers in vaccines promotes the mucosal immune response. Once in the host, they become valuable factories of molecules that act as natural adjuvants or specific antigens, harboring motifs sensed by mucosal APCs as danger signals that overcome oral tolerance. In general, the proinflammatory conditions favor the development of stronger local and systemic immune responses. Thus, appropriate adjuvants or delivery systems of antigens, or both, may critically promote the induction of protective mucosal responses (Chin'ombe, 2013; Ura et al., 2014; Lavelle and Ward, 2022). Accordingly, several mucosal vaccine strategies have been developed against flaviviruses, and their promising results have been documented. This review describes these approaches against the most relevant flaviviruses for human and animal health ordered by the number of publications and the type of platform used. They are classified as follows: direct antigen administration (inactivated and attenuated virus, subunits), non-replicant vectors, and replicant vectors. A graphical representation of these platforms is shown in **Figure 4**. A summary of the next section is presented in the **Supplementary Table 2**.

## 5. Mucosal Vaccine Approaches Used Against Flavivirus

### 5.1 DENV

#### 5.1.1 Subunits

Recombinant protein subunit vaccines are composed of at least one type of viral antigen produced in heterologous expression systems. Although significantly safer than attenuated and inactivated vaccines, they are less immunogenic (Wang et al., 2016b).

The approach proposed by Lazo-Vazquez et al. is a recombinant protein composed of the domain III of E protein (EDIII) from the four DENV serotypes and the C protein adjuvanted with oligodeoxynucleotide 39M. This tetravalent subunit was administered to mice three times by IN or intraperitoneal (IP) route, and both routes elicited neutralizing antibodies. Interestingly, the mucosal route favored the DENV-specific cell-mediated immunity (Lazo Vazquez et al., 2017).

To demonstrate the feasibility of the oral delivery, the subunit antigen made of EDIII from the four DENV serotypes was expressed in stably transformed lettuce chloroplasts. After gastrointestinal tract assays were conducted, the *in vitro* gastrointestinal digestion analysis showed that the antigen was well protected when passing through the oral and gastric digestion phases, but it was degraded during the intestinal phase. Furthermore, the antigen was immunogenic in rabbits when administered systemically (van Eerde et al., 2019).

##### 5.1.1.1 Fusion proteins

Different improvements can be made during the design of recombinant antigens by adding heterologous sequences (antigens from the same pathogen or a mixture with other pathogens, or even molecules with a specific function). The latter can guide the expression in a particular cell compartment (i.e., secreted vs intracellular), to act as adjuvant molecules, to modulate immune responses with interleukins, or to target specific cells (i.e., complement 5a receptor [C5aR]-bearing cells), among others functions. The combination of antigens of interest with heterologous sequences made by molecular biology has been named fusion or chimeric proteins. 

Kim et al. fused consensus EDIII from DENV to cholera toxin B subunit (CTB) and expressed this immunogen in transgenic rice calli (*Oryza sativa* L). Then, BALB/c mice were orally immunized four times with the lyophilized powder from the cell suspension cultures. DENV serotype-specific serum IgG responses were detected after the first boost, and they increased after the second one in animals immunized with the fusion protein. In contrast, no IgG responses were seen in groups immunized with EDIII alone or non-recombinant rice cells. Discrete but evident sIgA responses in feces were observed in mice treated with the fusion protein, while lesser responses were observed with EDIII alone. The antigen-specific lymphocyte stimulation in splenocytes confirmed the immunogenicity of the EDIII-CTB fusion protein expressed in transgenic rice given orally (Kim et al., 2016).

It is known that M cells, the specialized epithelial cells for transcytosis of luminal antigens in the PPs of the intestine, are a cellular target for the development of oral vaccines through the engagement of C5aR expression. Using this approach, Kim et al. evaluated peptide Co1, an M cell-targeting ligand. The peptide was cloned along with the DENV2 NS3 region aa 296–618. The resulting Co1-NS3 chimeric protein was expressed in *Escherichia coli* and administered orally to BALB/c mice twice at 2-week intervals. Four weeks later, splenocytes and PPs were obtained, and another subgroup was challenged with DENV2 (Kim et al., 2018).

The results showed that the frequency of IFN-γ producing CD8^+^ T cells and IFN-γ production in supernatants were increased in splenocytes and PP cells from animals either immunized or challenged after immunization with the fusion protein, versus those treated with NS3 alone or the phosphate-buffered saline (PBS) group. The authors concluded that Co1 may act as a mucosal vaccine adjuvant through C5aR, targeting M cells that induce mucosal CD8^+^ T cell response against DENV (Kim et al., 2018).

Nguyen et al. linked peptide Co1 to tetravalent tetrameric EDIII from all four serotypes of DENV; this chimeric protein was expressed in *Saccharomyces cerevisiae.* An *in vivo* antigen uptake assay in mice was carried out, and the results showed well-defined patches of overlapping sections expressing DENV antigens, M-cell specific lectin, and C5aR on the M cell surface when the EDIII-Co1 fusion protein was administered. Conversely, no interaction with M cells was detected when PPs were from animals administered with EDIII alone. The main assumption of this study is that affinity for M cells is a preliminary requirement for an effective oral vaccine, which was evinced through this experimental strategy (Nguyen et al., 2015).

A similar approach was explored by Yang et al., using an expression system with transgenic rice and an extra *in vitro* antigen uptake assay (Kim et al., 2013b). They took a gut loop containing PPs from a male BALB/c mouse and treated it with the protein extract from non-transgenic rice calli and transgenic rice expressing EDIII or EDIII-Co1 fusion protein. Both the *in vitro* and *in vivo* antigen uptake assays showed the binding of ligand EDIII-Co1 chimeric protein to M cells on PPs. No interaction was detected with EDIII alone or non-transgenic mice, pointing out the feasibility of this strategy as a potential mucosal vaccine against DENV (Kim et al., 2013b).

Kim et al. prepared an oral DENV vaccine tested in BALB/c mice by fusing an antigen EDIII of DENV2 to OmpH of *Yersinia enterocolitica*, a different ligand for C5aR in M cells, and analyzed the antibody and cellular responses. To test if oral priming induced systemic tolerance, some mice were boosted once with IP EDIII without ligand (Kim et al., 2013a).

The results showed statistically higher serum IgG and fecal sIgA responses in the mice immunized with the chimeric protein (EDIII-OmpH) or EDIII plus cholera toxin (CT)(positive control), in comparison with EDIII alone or PBS. Similar behaviors were observed in the number of EDIII-specific IgG and IgA-secreting cells, stimulation index, IL-4- and IL-6 secreting cells from PPs, and splenocytes determinations. The boosted animals showed higher serum IgG and fecal sIgA responses in EDIII + CT group vs EDIII and PBS control. Meanwhile, the fusion protein EDIII-OmpH was significantly enhanced only in fecal sIgA. Despite this, the neutralization activity in sera from the positive controls and the fusion protein was similar, as were the numbers of EDIII-specific IgG (splenocytes and PPs) and IgA (lamina propia)-secreting cells. Collectively, these results indicate that oral EDIII-OmpH successfully primed the humoral and cellular responses in systemic or mucosal compartments. The humoral response neutralized the DENV, the induced immune response originated from Th2-type cytokine-secreting cells, and these responses were not tolerogenic (Kim et al., 2013a).

#### 5.1.2 Nanoparticles

NPs are tiny particles made of several organic and non-organic materials. Their size ranges from 1 to 100 nm and they are biocompatible, biodegradable, and relatively easy to produce. Because of this, they are suitable candidates as delivery systems of nucleic acids, peptides, and proteins. They can protect the molecular cargo from enzymatic degradation, a key issue for mucosal vaccination, allowing to improve the bioavailability of the antigen to induce the innate and adaptive immune responses (Najahi-Missaoui et al., 2020).

Accordingly, approaches for mucosal DENV vaccines have been developed using NPs. Nantachit et al. used chitosan (CS) and trimethyl chitosan (TMC) NPs to carry DENV immunogen. The immunogen used in this approach was the domain III of DENV3 E protein (EDIII-D3) loaded into trimethyl chitosan NPs (EDIII-D3 TMC NPs). The *in vitro* model for nasal response used primary human nasal epithelial cells. The interaction between these cells and the NPs carrying EDIII-D3 induced proinflammatory cytokines (IL-1β, IL-6, TNF-α), type-I IFN, growth factors (GM-CSF, IL-7), chemokines (MCP-1, MIP-1β, IL-8), T-helper 1 (Th1)-related cytokines (IL-2, IL-12p70, IL-17, IFN-γ), and T-helper 2 (Th2)-related cytokine (IL-4). This *in vitro* assay suggested a potential mucosal delivery system for DENV proteins that can produce an antiviral immune response (Nantachit et al., 2016).

Subsequently, Vemireddy et al. describes that CS can be used to stabilize the emulsion of oleic acid-water. The design of this nanoemulsion allowed to activate the innate and adaptive immune responses and deliver the recombinant tetravalent DENV antigen. Mice immunized with the nanoemulsion bearing the tetravalent DENV antigen showed a robust antigen-specific humoral and cellular response. It was characterized by increased IgG1, IgG2a, and IgA titers; high concentrations of IFN-γ and IL-4; and increased rates of CD8^+^ T cells. In this process, the antigen cross-presentation and the sustained release of the DENV antigen by the emulsion play a significant role in the induction of the protective immune response (Vemireddy et al., 2018).

Consistent results were observed when this group used a cationic pH-responsive polycaprolactone NP as a delivery system for the recombinant tetravalent DENV antigen. In this study, hydrazine modified the polymer polycaprolactone, making it partially cationic and allowing its mucoadhesiveness. Once in the endolysosomal compartment, the NP can escape due to the protonation of free amines in the polymer. This characteristic enhances antigen cross-presentation. Cytotoxicity assays confirmed the safety of this NP. *In vitro* assays for antigen colocalization and cross-presentation have revealed its successful use as a tetravalent DENV antigen delivery system. The *in vivo* evaluation in BALB/c mice using recombinant DENV antigen for IN immunization showed that the modified polymer with 457 μM/mg of free amine groups effectively stimulated humoral (IgG, IgA, IgG2a/1 antibodies), and enhanced CD8^+^ T cells immune responses. The overall data suggest that the pH-responsive polycaprolactone NP is a versatile system for effective mucosal antigen delivery (Vemireddy et al., 2019).

#### 5.1.3 Bacterial Ghost

Since it has been described that developing a DENV vaccine is generally challenging, another interesting approach is the use of an empty bacterial cell envelope (bacterial ghost) that expresses a recombinant DENV antigen. Bacterial ghosts are Gram-negative bacterial envelopes produced by controlled expression of cloned gene E from a bacteriophage, forming a lysis tunnel structure within the envelope of the living bacteria. Bacterial ghosts have immune-stimulatory surface molecules, such as pathogen associated molecular patterns (PAMPs), lipopolysaccharides, and adhesins. They have been used as successful heterologous antigen delivery systems (Jalava et al., 2002), as demonstrated by Kim et al., who used a bacterial ghost from *Salmonella enterica* serovar Typhimurium to express the envelope protein E domain III (ST-EDIII) of each DENV serotype. The BALB/c mice were treated once orally with either the individual ST-EDIII constructs or a mix of all four ST-EDIII constructs, followed by the intramuscular (IM) administration of the purified EDIII protein. The results showed elevated titers of EDIII-specific IgG, IgG1, and IgG2a, and specific proliferative activity of CD3^+^CD4^+^ T-cell subpopulations. In addition, a significant reduction in the viral load was detected in the ST-EDIII vaccinated group after the challenge with DENV-infected K562 cells (Kim et al., 2020).

#### 5.1.4 Replication Vectors

Replicating vector vaccines include microorganisms that can still replicate inside or outside the cells and infect new cells that will also make the vaccine antigen. Among the replication vectors, several bacterial strains genetically modified are promising mucosal vaccine candidates. Some flavivirus antigens have been successfully expressed in live attenuated bacterial vectors as *S. enterica*, *Lactococcus lactis*, and yeast.

##### 5.1.4.1 Replicative Vector: Bacteria

The facultative anaerobic Gram-negative *S. enterica* is an important pathogen of animals and humans, causing a variety of infectious diseases. It has also demonstrated its potential as a live attenuated bacterial vector to carry heterologous antigens for vaccine purposes (Galen et al., 2016; Galen et al., 2021). After the oral administration and following the natural route of infection, *S. enterica* colonizes internal lymphoid tissues and remains there to continuously synthesize (as inner factories) and deliver recombinant antigens. These bacteria show tropism of APCs, allowing the induction of mucosal and systemic immune responses (antibody response and cell-mediated immune response) (Galen et al., 2016).

In 1990, Cohen et al. reported an attenuated *S. enterica* serovar Typhimurium carrying the plasmid that encodes E protein of DENV4 which is associated with the generation of neutralizing antibodies. These recombinant bacteria-induced antibodies recognized native DENV in mice (Cohen et al., 1990). A decade later, Liu et al. described a recombinant *S. enterica* serovar Typhimurium SL3261 strain expressing a secreted DENV2 NS1 and *Yersinia pestis* F1 (Caf1) fusion protein (rNS1:Caf1). The oral immunization of mice with 1×10^9^ cfu of these recombinant bacteria induced low levels of NS1-specific antibody response and failed to protect mice after a DENV challenge. However, the approach where mice received parental NS1 protein followed by an oral *Salmonella* boosting protocol enhanced the NS1-specific serum IgG response and the protective efficacy. The authors observed better results when they administered the antifungal antibiotic amphotericin B (AmpB) as adjuvant (Liu et al., 2006).

**Figure 5:**
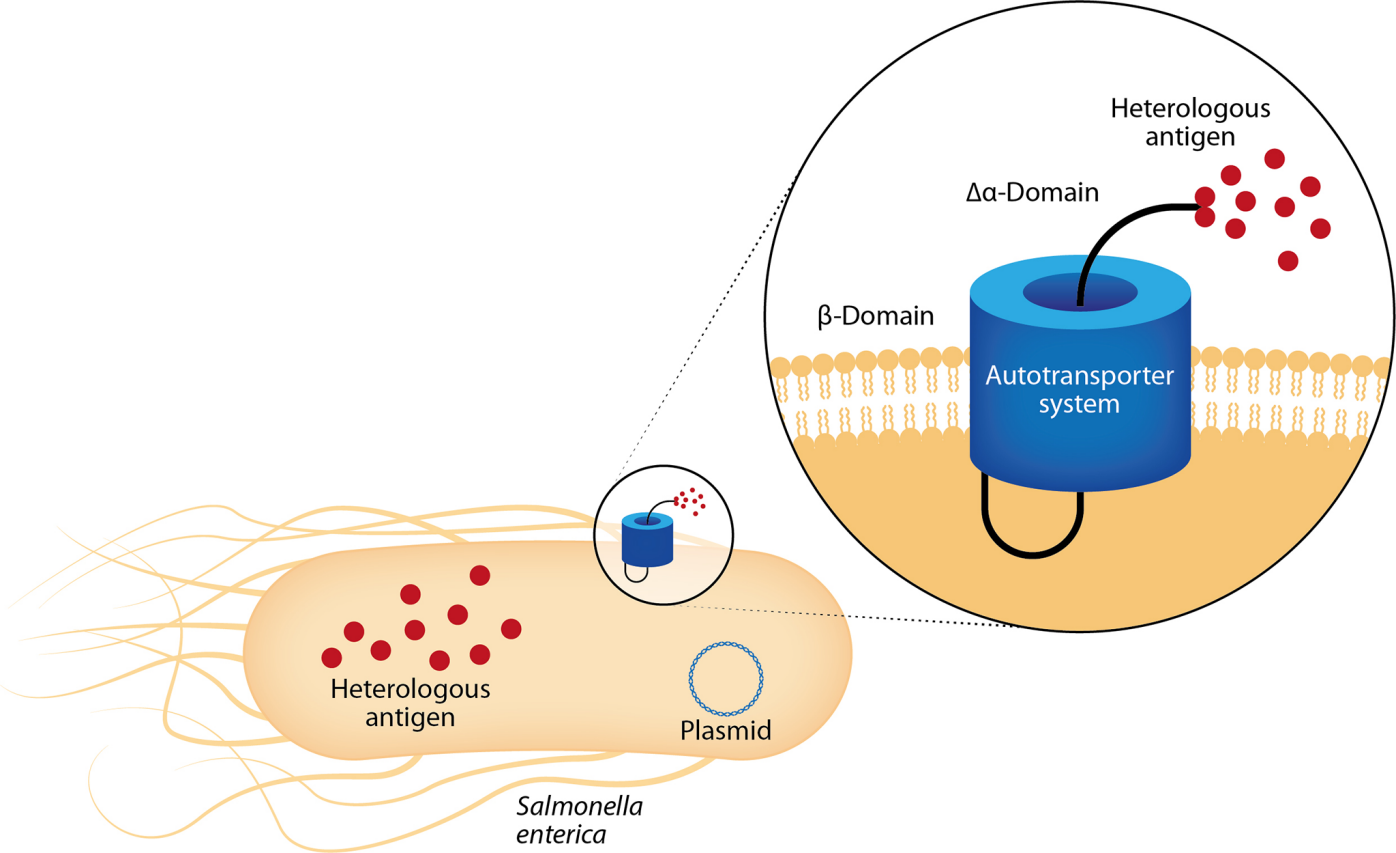
Mucosal vaccines with bacterial replication vectors: *Salmonella enterica* as delivery system. After mucosal administration, live-attenuated *S. enterica* colonize lymphoid tissues and remain there, serving as factories that continuously synthesize and deliver heterologous antigens. These bacteria show tropism for antigen-presenting cells, allowing the induction of mucosal and systemic immune responses. They successfully carry heterologous antigens, as genes and proteins, in the cytosol or can display them on its surface through autotransporter systems as MisL protein. The sequence encoding the MisL α-domain is genetically modified to add the sequence encoding the heterologous antigen.

More recently, Luria-Perez et al. reported an attenuated *S. enterica* serovar Typhimurium SL3261 that expresses a fusion protein on its surface through the domain of the autotransporter MisL. Starting from the N-terminal, the fusion protein contains a fusogenic sequence that disrupts membranes, a CTL epitope (NS3 protein 298–306-amino acid from the DENV2), a molecular tag, and a recognition site for the protease OmpT to release the peptide to the milieu. After the oral administration, the recombinant *Salmonella* strains expressing the fusogenic DENV peptide showed specific proliferative responses in the murine model and elicited CTL responses against the NS3 protein. This was demonstrated by *in vitro* and *in vivo* assays (Luria-Perez et al., 2007). The autotransporter system has been used in several protocols to express or release the recombinant proteins from the surface of enterobacteria (**Figure 5**) to induce a strong humoral response against heterologous antigens (Ruiz-Perez et al., 2002; Ruiz-Olvera et al., 2003; Pompa-Mera et al., 2011). Luria-Pérez et al. used this system to express a peptide from EDIII antigen from DENV2 on the surface of *S. enterica* as a vaccine candidate for DENV2 (manuscript in preparation).

Recombinant *L. lactis* is an aerobic Gram-positive bacteria found in the intestine of most animals, including humans. Its potential use as a mucosal delivery vehicle for mucosal flavivirus vaccines has been extensively investigated (Pontes et al., 2011). Recently, Sim et al. reported a recombinant *L. lactis* strain producing the EDIII antigen from DENV2. The ability of these live recombinant bacteria to trigger a systemic anti-EDIII IgG antibody response in mice upon nasal or oral administration showed that the high antibody anti-EDIII response depended on the administration route. *In vitro* assays showed that sera from the orally immunized mice had the highest activity when neutralizing the infection by DENV (Sim et al., 2008).

##### 5.1.4.2 Replicative Vector: Yeast

As technology has moved forward, new vaccine opportunities have also been created. Recently, a study by Bal et al. used a murine model treated orally with recombinant whole yeast cells (WC) or cell-free extract (CFE) containing recombinant *E. coli* heat-labile toxin protein B-subunit (LTB) fused to the consensus DENV EDIII, LTB-scEDIII. They showed that mice immunized with WC or CFE LTB-scEDIII stimulated a systemic humoral immune response in the form of DENV-specific serum IgG as well as a mucosal immune response in the form of sIgA. Sera obtained after both oral administrations successfully neutralized DENV1. However, the overall results suggest that the LTB-scE-DIII fusion protein delivered in CFE rather than WC is a promising and potent oral vaccine candidate against DENV infection (Bal et al., 2018b). Better results were reported by the same group using yeast surface display technology. They developed an oral dengue vaccine candidate using whole recombinant yeast cells expressing the recombinant fusion protein of M cells targeting ligand Co1 fused to the synthetic consensus DENV envelope domain III (scEDIII). Female BALB/c mice were orally immunized with recombinant yeast cells. The surface-displayed Co1-scEDIII-AGA yielded a better systemic humoral immune response in the form of DENV-specific serum IgG and a mucosal immune response as sIgA vs non-displayed Co1-scEDIII. Moreover, a long-term memory analysis performed after the last oral immunization showed that Co1-scEDIII-AGA-fed mice showed a significant immune response, both humoral and mucosal, upon the IP booster dose of alum-adsorbed purified *E. coli*-expressed scEDIII (Bal et al., 2018a).

The number of mucosal vaccine developments against DENV is the highest among flaviviruses, most likely due to its epidemiological and economic impact worldwide. The oral route has been tested more often than the IN route, and recombinant subunits were the vaccine platform of choice, mainly as fusion proteins, and the replication vectors (bacteria and yeasts).

Mucosal vaccination using *S. cerevisiae* as a delivery system has advantages since this yeast is generally recognized as safe by the FDA and has long been used as a food supplement. Furthermore, the carbohydrate molecules on the cell wall of yeast cells act as PAMPs. Transgenic plants, especially rice, are another feasible vaccine platform that might eventually achieve a similar status. This approach is applicable to all flaviviruses.

All oral vaccines have to overcome the aggressive environment in the gastrointestinal tract (low pH and intestinal proteases) to reach the gut-associated lymphoid tissues, chemically and physically protected by barriers preventing efficient uptake. Furthermore, these vaccines have to overcome the tolerogenic responses present in the gut. In spite of this, several experimental approaches have been tested to improve immunity by the oral route. In this context, specific regions from a flavivirus can be linked to molecules that improve immunogenicity. For instance, these antigens have been fused to intestinal toxins as adjuvants, or have been targeted to M-cells, which enhance the mucosal and systemic immune response against DENV induced by oral vaccination. This can be extrapolated to other flaviviruses.

### 5.2 JEV

#### 5.2.1 Inactivated Vaccines

Inactivated vaccines take the whole virulent virus, or one very similar to it, and inactivate it using chemicals, as formalin or glutaraldehyde, or physical methods like heat or radiation. These vaccines only contain structural proteins, so they are unable to replicate. They render a less broad immune response in comparison to attenuated or live vaccines. Sufficient titers are difficult to manufacture for preparations, while the cost per dose is higher, and multiple immunizations are commonly required. Still, inactivated vaccines are safer since they cannot revert to the virulent phenotype (Vaughn et al., 2009).

A mouse brain-derived formalin-inactivated JEV vaccine was co-administered with either killed *Bordetella pertussis* or two adjuvants from bacteria (CT and pertussis toxin) to mice by IN, oral, and transcutaneous routes. Overall, the best results were achieved with the IN route. When tested alone, the inactivated vaccine provided a robust neutralizing antibody response. The response was not increased when co-administered with killed *B. pertussis*, unlike what was observed with bacterial toxins. On the contrary, the ideal response was obtained when mixed with toxins. The IN immunization of the inactivated vaccine and bacterial adjuvants showed an antibody response similar to a parenteral immunization regime but with better immune cellular responses (Harakuni et al., 2009).

#### 5.2.2 Subunits

Wang et al. reported the oral administration of leaf extracts from transgenic rice expressing the E protein of JEV as part of the food (5 times/week/1 month) given to BALB/c mice. The animals showed evident systemic (IgG in serum) and mucosal (IgG and IgA in the intestinal wash) immune responses vs the group immunized with non-related transformed rice. Similar results were achieved in animals fed with the E recombinant immunogen obtained from *E. coli*, demonstrating the feasibility of oral vaccination against JEV through food (Wang et al., 2009).

#### 5.2.3 NPs

JEV vaccines have also benefited from NPs approaches. Dumkliang et al. used attenuated Japanese encephalitis chimeric virus vaccine (JE-CV)-loaded mucoadhesive NPs based on CS or chitosan maleimide (CM) as a novel mucoadhesive polymer with antigen-uptake properties. After IN immunization of the murine model, the results revealed a successful protection, an enhanced concentration of IFN-γ, and higher titers of antigen specific sIgA levels compared to mice immunized with a subcutaneous injection. The latter group also showed high rates of protection and cytokines but failed to induce a mucosal response measured by sIgA titers. These observations show the promising approach of IN vaccination with NPs as an alternative route for JE protection due to the stimulatory effects on both mucosal and systemic immune responses (Dumkliang et al., 2021).

##### 5.2.3.1 Liposomes

Liposomes are spherically shaped microscopic vesicles that consist of one or more phospholipid bilayer membranes. They have the properties of a nano-scale, biofilm similar structure, and are excellent as a delivery system of several molecules. Their aqueous phase can contain hydrophilic drugs, and their phospholipid bilayer can localize lipophilic drugs (Li et al., 2019).

Liposomes are another novel oral vaccine approach for JEV. Lin et al. recently described a liposome made with di-stearoyl phosphatidylcholine and cholesterol, bearing JEV NS1 protein (Lip-JENS1). A single oral immunization of a murine model with Lip-JENS1 elicited a low detectable serum NS1-specific IgG antibody response. However, after adding amphotericin B (AmpB) as an adjuvant (using very low amounts vs higher amounts to obtain antifungal effect), an enhanced systemic antigen-specific antibody response was observed, providing excellent protection against lethal JEV challenges. This study also documented high IL-1b, IL-6, and TNF-α expression levels in PP lymphocytes (PPL) of AmpB-treated mice. These results suggest that the oral administration of the Lip-JENS1 liposome with AmpB represents a potential mucosal vaccine to protect against JEV infection (Lin et al., 2010).

#### 5.2.4 Replication-Defective Viral Vectors

Replication-defective viral vectors are another proven technology used to display flavivirus antigens for vaccine purposes. Among them, adenoviruses have been commonly used for gene transfer experiments given their ability to infect a wide group of different cell types. They can harbor large genes in their genome incorporated *via* homologous recombination techniques (Sakurai et al., 2022). Studies by Appaiahgari et al. reported a replication-defective human adenovirus type-5 (rAd5) platform that expresses and releases the PrM and E proteins of JEV. The recombinant virus RAdEa synthesized Ea, the membrane-anchored E protein, while RAdEs synthesized Es, the secretory E protein. After the oral immunization of BALB/c mice with RAds, low titers of anti-JEV and less JEV neutralizing activity were observed as compared with mice immunized by IM route with RAds. These IM-immunized mice showed high titers of anti-JEV antibodies with neutralizing properties, specific cellular immune response against JEV, and also a complete protection against a lethal dose of JEV given intra-cerebrally (Appaiahgari et al., 2006).

Better results were reported by Li et al., who constructed a rAd5 expressing immunodominant epitopes against JEV. The multiple-epitope (TEP) gene of JEV was designed with a length of 564 bp, corresponding to the (60–68) - (327–333) - (337–345) - (373–399) - (397–403) - (436–445) aa sequence of E protein based on the JEV SA14 strain. The BALB/c mice groups were immunized with the IM or oral recombinant adenoviruses twice at 2-week intervals with doses ranging from 1×10^7^ to 1×10^8^ TCID_50_. Higher antibody titers were obtained in mice that were orally immunized when higher doses of rAd5-TEP were used. However, the IM immunization of mice with rAd-TEP generated more significant titers of anti-JEV antibodies and JEV neutralizing activity than the oral injection. The highest level of cell-mediated immune responses produced by IM immunization was also documented (Li et al., 2008).

The works cited in this section used a variety of platforms to develop a mucosal JEV vaccine, from the classical inactivated one to a recombinant rAd5 created by molecular biology. Although better results were achieved with a parenteral route (IM) instead of a mucosal route for JEV vaccines, it is worth mentioning that it happened mainly when rAd5 was used as a vector. Conversely, IN vaccination was the best option to get better immune cellular responses when an inactivated vaccine was mixed with bacterial adjuvants, highlighting the importance of the combination of the delivery system, and the use of adjuvants in the induction of local and systemic immune response when mucosal vaccines are used.

### 5.3 WNV

#### 5.3.1 Subunits

Fassbinder-Orth et al. used a *Drosophila* expression system to obtain recombinant E protein from WNV. Three doses of a nontoxic mutant form of *E. coli* heat-labile enterotoxin (LT), LTK63, along with the antigen were administered to chickens (*Gallus gallus*) orally or intramuscularly. Two weeks after the final vaccination, the animals were subcutaneously challenged with a crow isolate of WNV. The results showed higher levels of viremia in animals vaccinated orally in comparison with those that received IM doses, despite higher immunoglobulin M (IgM) production in oral route at day 21 post-infection. Anti-E IgY production was detected in animals immunized intramuscularly even before the challenge, while no detection was recorded in the rest of the groups on the same day. In this study, IM vaccination of E protein from WNV plus LTK3 yielded better results than its oral equivalent (Fassbinder-Orth et al., 2009).

Exploring the adjuvanticity of five mast cell-activating compounds (MCAC), the DIII of WNV E protein was used as an IN immunogen administered three times to BALB/c mice. The animals were challenged intraperitoneally with a WNV strain. Four out of five MCAC induced evident EDIII-specific serum IgG titers (10^3^–10^4^). In contrast, animals immunized only with the recombinant subunit vaccine showed undetectable levels. The challenge results demonstrated that the protection by MCAC ranged from 42 to 75%. Meanwhile, the positive adjuvanticity control was 100%, and EDIII alone showed 33%. The authors used a mucosal subunit vaccine from WNV to demonstrate the induction of a specific and protective immune response when this immunogen was adjuvanted to MCAC (Johnson-Weaver et al., 2021).

#### 5.3.2 Fusion Protein

Tinker et al. studied the effect of an IN fusion protein composed of DIII from WNV linked to non-toxic CT CTA_2_/B domains as an adjuvant on the immunogenicity of BALB/c mice. The DIII-CTA_2_/B chimera elicited the highest serum antigen-specific IgM, IgG, and IgA titers vs the rest of the groups. The increased IgG2a/IgG1 ratio of the chimeric protein and mixed antigen plus adjuvant indicated a Th1-type immune response. The induced humoral response was functional since the antigen plus CTA2/B can activate the complement *in vitro*, and showed the highest bactericidal activity. Additionally, in the absence of adjuvant, the DIII antigen was also effective in stimulating significant systemic IgG responses triggered by the increased dosage. The results from this platform justify further investigations using WNV chimeric recombinant subunit vaccines (Tinker et al., 2014).

#### 5.3.3 NPs

Alginate, a natural polysaccharide used to encapsulate controlled-release substances, was combined with spermidine to microencapsulate a DNA vaccine encoding PrM and E glycoproteins from WNV. The oral or IM preparation was administered once to captured fish crows (*Corvus ossigrafus*). Six weeks after the vaccination, the birds were subcutaneously challenged with the WNV 397-99 strain. The oral administration did not elicit neutralizing antibodies, although it partially protected the animals after the challenge (50% of survival). Meanwhile, the IM administration provided complete protection and was associated with reduced viremia (Turell et al., 2003).

#### 5.3.4 Viral Vectors

In the list of replication vectors, viral vectors also represent a promising platform for a flavivirus mucosal vaccine. They can express heterologous antigens and induce antigen-specific cellular and humoral immune responses without the need for adjuvants (Humphreys and Sebastian, 2018).

Iyer et al. documented a recombinant Vesicular Stomatitis virus (VSV)-based WNV vaccine. They constructed recombinant VSVs specifying either the Indiana or Chandipura virus G glycoprotein and expressing the WNV E glycoprotein. After IN immunization of the mouse model with Indiana (prime) or Chandipura (boost), the animals were challenged with virulent WNV-LSU-AR01 and 90% of the vaccinated mice survived. The immunological analysis revealed a strong neutralizing antibody response against WNV and a robust cellular immune response, evidenced by the presence of CD4^+^ CD154^+^ IFN^+^ T cells and CD8^+^ CD62L^low^ IFN^+^ cells, while regulatory T cells were downregulated (Iyer et al., 2009).

Wang et al. reported another WNV mucosal vaccine by developing a recombinant and virulent Newcastle disease virus (NDV) La Sota strain expressing WNV pre-membrane/envelope (PrM/E) proteins (rLa-WNV-PrM/E) and evaluating its immunogenicity in mammals and poultry. Three weeks after the oral or nasal immunization with a 5×10^8^ EID_50_ of recombinant rLa-WNV-PrM/E, the chickens, ducks, and geese received a boosting protocol. Significant levels of WNV-specific IgG were documented, the same as those observed in animals that had received the IM rLa-WNV-PrM/E (Wang et al., 2016a).

Even though most of the flavivirus reviewed here constitute a threat to human health, WNV highlights the need to expand the vaccination effort to animals as well since some birds and horses can be infected by mosquito bites. Studies have used bird animal models and others have included mice to complete the immunogenicity data, showing that oral and IN routes would promote the massification of vaccination protocols in birds at affordable costs.

### 5.4 ZIKV

#### 5.4.1 Attenuated Vaccines

Attenuated vaccines are made up of an active live virus modified to weaken and reduce its virulence. These wild-type (WT) viruses are attenuated *in vitro*, usually by repeated culturing (Yadav et al., 2014). Meanwhile, recombinant attenuated vaccines are generated using molecular biology techniques which attenuate a viral strain to carry the gene(s) encoding the desired viral antigen. These vaccines have several attractive features, including the ability to stimulate both humoral and cell-mediated immunity (Girard and Koff, 2013); however, in some cases, they could revert to the virulent phenotype.

In a particular mouse model with a deficient type I IFN innate immune system, Martinez et al. studied the effect of rectal vs subcutaneous infection with ZIKV strains. The WT ZIKV PRVABC59 strain was used for the subcutaneous challenge of immunized animals, while the ZIKV PRVABC59 mutant strain was used as an attenuated vaccine (Martinez et al., 2020).

The first part of the study compared both infection routes with WT ZIKV, resulting in the death of animals in the subcutaneous route and 100% survival rate in animals infected rectally. Higher viral loads were detected in animals infected subcutaneously in comparison with those infected rectally (Martinez et al., 2020).

The second part of the study consisted of animals primed with ZIKV strains and challenged subcutaneously. The survival in animals immunized with the attenuated strain was 80%, compared to 100% in animals primed with WT and 40% in unprimed animals. The cellular response in these groups showed a significantly higher percentage of DC and T cells in spleens from animals treated with the attenuated strain, in comparison with cells from animals with the WT strain (Martinez et al., 2020).

These results showed that a high dose of subcutaneous ZIKV rendered an accelerated systemic infection and acute neuropathological outcome. In contrast, rectal ZIKV led to subclinical, non-neurological disease outcomes. Then, the mucosal priming with ZIKV strains could provide protective immunity. This work encourages further research of mucosal ZIKV immunization to prevent future outbreaks (Martinez et al., 2020).

#### 5.4.2 Fusion Protein

Márquez-Escobar et al. constructed a chimeric protein with three epitopes from ZIKV E protein and LTB, a sequence expressed in the marine microalgae *Schizochytrium* sp. The mice (BALB/c) received four oral or subcutaneous doses of the intact microalgae biomass or soluble protein extract, respectively. The results showed that recombinant microalgae elicited significantly higher serum IgG responses for each one of the three epitopes from ZIKV or LTB. A similar behavior was produced by IgA from feces, directed to one of the epitopes and the LTB fraction. The work concluded that this fusion protein expressed in microalgae was immunogenic and induced either systemic or mucosal responses when administered orally (Marquez-Escobar et al., 2018).

The formyl peptide receptor-like-1 inhibitory protein (FLIPr) is an FcγR antagonist secreted by *Staphylococcus aureus* linked to the ZIKV domain III (ZEIII-FLIPr). Hsieh et al. offered a proof-of-concept to demonstrate that IN FLIPr could enhance immunogenicity in mice and improve protection against infection. Mice (immunocompromised AG129 lacking the receptor for types-I and II IFNs, IFNs α/β/γ) were immunized three times and received challenged IP. There were significantly higher titers of neutralizing systemic antibodies (serum IgG and IgA), or mucosal antibodies (vaginal lavages and bronchoalveolar fluid IgA) in mice vaccinated with the fusion protein, compared to those immunized with EIII antigen or treated with PBS. The viremia titer in the challenge experiments was the lowest and showed the longest survival times in mice that were administered with rZEIII-FLIPr. The authors suggested that IN rZEIII-FLIPr is a potential vaccine candidate against ZIKV (Hsieh et al., 2021).

#### 5.4.3 Replication-Defective Virus

Recent studies by Steffen et al. have documented the efficacy of a mucosal vaccine against ZIKV. Groups of C57BL/6J mice received IN immunization of 3×10^7^ human adenovirus type 5 viral particles expressing PrM and E proteins of ZIKV. The results showed the induction of both cell-mediated and humoral immune responses to ZIKV epitopes. They found that the antigen-specific CD8^+^ T cell against dominant ZIKV T cell epitope plays a major role in the protection against a ZIKV challenge (Steffen et al., 2020).

Given that ZIKV is the only flavivirus transmitted sexually through mucosae, mucosal immunization is the most justifiable type of vaccination. One of the examples reviewed here is mainly a proof-of-concept (Martinez et al.) since the authors studied the effect of the rectal route of infection with WT and attenuated ZIKV strains, but obviously this is not a practical route of immunization for animals, much less for humans. To circumvent this, more feasible routes (oral and IN) have been explored, and some have yielded encouraging protection results.

### 5.5 TBEV

#### 5.5.1 Replicative Vector: Virus

TBEV mucosal vaccines have also been reported by Ryzhikov et al. They use a recombinant TK- variant of the vaccinia WR strain carrying genes that encode for structural and nonstructural proteins of TBEV. After the IN immunization of mice with this recombinant vaccine, a mucosal and humoral immune response was induced, and protection against TBEV challenge was observed. It is worth mentioning that the IN immunization induced more favorable results compared with mice immunized by scarification and subcutaneous administration (Ryzhikov et al., 1998). Overall, these results suggest that mucosal viral-vectored vaccines can efficiently induce protective humoral and cellular immune responses against flavivirus infections.

In summary, besides the work mentioned previously by Ryzhikov et al., there are some other successful vaccine approaches against other flaviviruses that achieved similar or even better results than the parenteral routes (Harakuni et al., 2009; Lazo Vazquez et al., 2017; Martinez et al., 2020; Dumkliang et al., 2021). Therefore, worthwhile to make more efforts to deep into the mucosal vaccination approaches for flaviviruses.

#### 5.5.2 Combined Strategies

Given that a single platform for mucosal immunization is not enough to elicit a robust immune response, researchers have explored combining different platforms as an effort to obtain better results.

Synthetic peptides in vaccination are based on specific immunodominant peptides, produced synthetically, from the original antigens that induce B-cell and/or T-cell responses. This is a safe strategy because the researchers do not need to work with whole pathogens, and the peptides are well characterized and are stable but they are poorly immunogenic.

The use of peptides as a vaccine platform for flaviviruses mucosal immunization has been explored in combination with an inactivated vaccine directed to TBEV. Goncharova et al. sought specific regions in the E protein of TBEV to avoid autoimmunity. They were obtained by informatics, rendering a 31-aa peptide (antigenic peptide 89-119) residue. In contrast, the strain Sofjin of TBEV isolated from the human brain was inactivated with formalin. These two immunogens were encapsulated into 200-400-nm NPs for IN administration to BALB/c mice. The positive control was an inactivated, subcutaneous commercial vaccine. After four doses, the groups received IP challenge with strain Sofjin of TBEV (Goncharova et al., 2006).

The most relevant result from this study is the complete protection against systemic challenges obtained with IN, inactivated vaccines. A peptide vaccine even achieved 58% protection. These observations indicate promising perspectives for further developments (Goncharova et al., 2006).

DNA immunization has been tested in combination with others against flavivirus. This platform works by injecting genetically engineered plasmids containing the DNA sequence encoding the antigen(s) against which an immune response is sought. Hence, the cells directly produce the antigen, triggering a protective immunological response (Hobernik and Bros, 2018). It is a safe, affordable strategy, yet it is not as immunogenic as a whole virus or recombinant proteins.

Goncharova et al. also compared four delivery systems carrying TBEV antigens. The first system, artificial virus-like microparticles (VLP), consisted of polyglycan-spermidine complexes covering pcDNA3/E-TBEV DNA. The second system was cationic liposomes with pcDNA3/E-TBEV DNA. The third system was an attenuated *Salmonella* strain containing pcDNA3/E-TBEV. The fourth system was a recombinant vaccinia strain with inserted genes of C, PrM, E, NS1, NS2a, NS2b, and NS3 proteins of TBEV. The results showed a Th1-type immune response in the BALB/c mice immunized with IN recombinant vaccinia-TBEV strain and VLP-pcDNA3/E-TBEV. The mice immunized with IN recombinant vaccinia-TBE strains were fully protected against the IP challenge with strain Sofjin of TBEV. In contrast, the inactivated TBEV vaccine failed to induce a significant level of protection (Goncharova et al., 2002).

Since there are some neurotropic flaviviruses (JEV, WNV, and TBEV), IN immunization is one of the most studied options to induce mucosal and systemic immunity. It aims to block the viral propagation into the brain through the olfactory pathway and neutralize virus multiplication in visceral organs. This was evident in several works that use this route, demonstrating IN vaccination is a likely alternative given the protection during the challenges with flaviviruses that cause encephalitis.

### 5.6 DTMUV

#### 5.6.1 Replicative Vector: Bacteria

Huang et al. used DNA vaccines vectorized with *S. enterica* serovar Typhimurium expressing C, PrM, and E proteins from DTMUV. Using this, ducks were immunized twice by oral route, then they were challenged intravenously with a DTMUV strain. A discrete but evident anti-viral IgY response was observed in animals orally immunized with recombinant *Salmonella*, and this response was neutralizing. The last finding corroborates the result of the challenge experiments, since the survival rate was 30% higher than the one observed in negative controls (Huang et al., 2018a; Huang et al., 2018b).

### 5.7 LIV

#### 5.7.1 Replicative Vector: Virus

Semliki Forest virus has also been analyzed as a viral vector for flavivirus mucosal vaccines. Fleeton et al. reported recombinant Semliki Forest virus (rSFV) particles encoding the PrME (rSFV-PrME) or NS1 (rSFV-NS1) proteins of LIV. Mice immunized with IN rSFV particles produced antibodies against PrME and NS1, mostly IgG2a. This indicates that a Th1 immune response was induced and a specific T-cell proliferative response to LIV antigens was observed. In challenge experiments, mice that had received IN vaccination with rSFV-PrME particles were protected against a fully virulent LIV strain (LI/31). Still, they were not protected against the challenge with a less virulent LI/I, a LIV strain. Mice that received IN immunization with rSFV-NS1 showed protection against LIV strains LI/31 and LI/I. Better results were observed in mice immunized with rSFV-PrME and/or rSFV-NS1 by IP route because they were significantly protected from a lethal IP challenge using two LIV strains (virulent LI/31 or less-virulent LI/I) (Fleeton et al., 1999).

DTMUV and LIV are the least relevant flavivirus in terms of human public health. However, they are potential models to study the immunogenicity elicited by candidate vaccines.

## 6. Conclusion

Given that one single platform will unlikely meet all the ideal requirements, vaccinologists must search for the continuous improvement of vaccines. Flaviviruses are constituted by sets of viruses with different characteristics in terms of infection, pathology in different systems (i.e., central nervous system), and economic issues. For example, ZIKV is the only sexually transmitted arbovirus while a group of flaviviruses causes encephalitis (JEV, WNV, and TBEV). In addition, dengue creates direct and indirect costs from its treatment and is the second largest arthropod-transmitted pathogen worldwide. The infection by WNV requires an expensive approach for its control and eradication since poultry is an amplifier host. Therefore, more affordable and friendlier alternatives for children and adult vaccination are necessary, considering that each case demands a specific vaccination approach.

To address the challenges in vaccination against flaviviruses, we propose mucosal vaccination as a potential option given its low reactivity, reduced costs, simple application, and non-invasive administration. Additionally, it requires no equipment nor specially trained health personnel for its administration. This type of vaccine prevents blood-borne infections because it is needle-free. Unlike parenteral inoculation, mucosal vaccination is an excellent option for mass application because it does not generate large amounts of biohazardous waste.

The works reviewed here show that mucosal vaccination elicits broad mucosal and systemic immune responses against flaviviruses. In this regard, oral and IN routes are the most common paths of administration in comparison with intravaginal and rectal routes. The latter ones are less practical for vaccine administration but they are important, considering that ZIKV can be sexually transmitted through mucosae.

Some studies reviewed here compare mucosal with parenteral immunization routes, revealing the latter showed higher humoral, cell, and protective responses. Still, better yields in protection data were achieved in mucosal routes than the parenteral ones in certain models. Another set of works used mucosal routes by testing several vaccination approaches, demonstrating strong immunogenicity and protection against flaviviruses. Overall, these studies documented the advantages of mucosal vaccination, encouraging the improvement of the current flaviviruses vaccines. This can be enhanced using the appropriate mucosal route of immunization, adjuvants, and delivery systems of antigens.

Mucosal vaccination against flavivirus and other pathogens causing infectious diseases in general is characterized by the ease of immunogen delivery, especially by the oral route. This constitutes a major advantage for the potential implementation of large-scale vaccination programs. Moreover, due to the needle-free method, it is a friendly route when compared with parenteral inoculation ones. Therefore, further research exploring simple or combined mucosal vaccination strategies must be performed to expand their use beyond flaviviruses to cover infectious diseases in general.

## Author Contributions

RL-P and GM-S contributed to the conception, design, and drafting of the manuscript. LS-V and PM-L contributed to data collection and manuscript drafting. PM-L contributed to the design of figures. GM-S contributed to the edition. All authors contributed to the article and approved the submitted version.

## Funding

This study received funding from Hospital Infantil de México “Federico Gómez” to RL-P Fondos Federales HIM-2018-080 SSA 1543. Basic Science project CB-2014-01#238736 was granted to GM-S, and PM-L received the predoctoral scholarship #711710, both from Consejo Nacional de Ciencia y Tecnología (CONACyT).

## Conflict of Interest

The authors declare that the research was conducted in the absence of any commercial or financial relationships that could be construed as potential conflicts of interest.

## Publisher’s Note

All claims expressed in this article are solely those of the authors and do not necessarily represent those of their affiliated organizations, or those of the publisher, the editors and the reviewers. Any product that may be evaluated in this article, or claim that may be made by its manufacturer, is not guaranteed or endorsed by the publisher.
