# Supplementary material for: Mucosal Vaccination: A Promising Alternative Against Flaviviruses
Source: Front Cell Infect Microbiol. 2022 Jun 15;12:887729. doi: 10.3389/fcimb.2022.887729 (PMC9241634; doi:10.3389/fcimb.2022.887729)
Supplement: Supplementary file 1 [file Table_1.docx]

Supplementary Table 1. Human vaccines against flavivirus, licensed or in clinical development

| **Virus** | **Vaccine candidate** | **Platform** | **Developer** | **Current Stage** | **Reference** |
| --- | --- | --- | --- | --- | --- |
| YFV | XRX-001 | Inactivated whole-virus | Xcellerex | Phase I | ([Monath et al., 2011](#_heading=h.26in1rg)) |
|  | YF-Vax (Stamaril) | Live-attenuated | Sanofi Pasteur | Licensed | ([World Health Organization, 2013](#_heading=h.1y810tw)) |
|  | vYF01 | Live-attenuated | Sanofi Pasteur | Phase I | NTC04142086 |
| DENV | TDENV-PIV | Inactivated whole-virus | GSK, Fiocruz & WRAIR | Phase I | ([Thomas and Rothman, 2015](#_heading=h.2jxsxqh)) |
|  | CYDTYD (Dengvaxia) | RLA | Sanofi Pasteur | Licensed | ([Sridhar et al., 2018](#_heading=h.44sinio)) |
|  | TV003 (Tetravax) | RLA | NIAID | Phase III | ([Kirkpatrick et al., 2016](#_heading=h.2s8eyo1)) |
|  | TAK-003 (DENVax) | RLA | Takeda | Phase III | NCT02747927 |
|  | V180 | Recombinant subunit | Merck | Phase I | ([Manoff et al., 2015](#_heading=h.3rdcrjn)) |
|  | TVDV | DNA vaccine | U.S. Naval Medical Research Center | Phase I | ([Beckett et al., 2011](#_heading=h.30j0zll)) |
| JEV | IC51 (IXIARO, JESPECT JEEV) | Inactivated whole-virus | Intercell AG | Licensed | ([Schuller et al., 2009](#_heading=h.1ksv4uv)) |
|  | JEBIK V | Inactivated whole-virus | BIKEN | Licensed | ([Kikukawa et al., 2012](#_heading=h.4d34og8)) |
|  | KD-287 (ENCEVAK, JEIMMUGEN INJ.) | Inactivated whole-virus | Kaketsuken | Licensed | ([Rupp et al., 2015](#_heading=h.lnxbz9)) |
|  | SA1414-2 | Live-Attenuated | CDIBP | Licensed | ([Bista et al., 2001](#_heading=h.1fob9te)) |
|  | JE-CV (ChimeriVax-JE, IMOJEV) | RLA | Sanofi-Pasteur | Licensed | ([Chin and Torresi, 2013](#_heading=h.3znysh7)) |
| WNV | HydroVax-001 | Inactivated whole-virus | OHSU/ NIAID | Phase I | NCT02337868 |
|  | Inactivated WNV | Inactivated whole-virus | Baxter Bioscience | Phase I, II | ([Barrett et al., 2017](#_heading=h.gjdgxs)) |
|  | rWN/DEN4Δ30 | RLA | NIAID | Phase I | ([Durbin et al., 2013](#_heading=h.3dy6vkm)) |
|  | ChimeriVax-WN02 | RLA | Sanofi Pasteur | Phase II | ([Dayan et al., 2012](#_heading=h.2et92p0)) |
|  | HBV-002 | Recombinant subunit | Hawaii Biotech | Phase I | NCT00707642 |
|  | VRC 302/VCR 303 | DNA | Vical/NIAID | Phase I | ([Ledgerwood et al., 2011](#_heading=h.17dp8vu)) |
| ZIKV | ZPIV | Inactivated whole-virus | WRAIR/NIAID | Phase I | NCT03008122 |
|  | VRC5283/ VRC5288 | DNA | NIAID | Phase II | NCT03110770 |
|  | GLS-5700 | DNA | Inovio GeneOne | Phase I | NCT02887482 |
| TBEV | FSME-Immun (TicoVac) | Inactivated whole-virus | Pfizer | Licensed | ([Who, 2011](#_heading=h.3j2qqm3)) |
|  | Encepur | Inactivated whole-virus | GSK | Licensed | ([Demicheli et al., 2009](#_heading=h.tyjcwt)) |
|  | Encevir | Inactivated whole-virus | Microgen/ Russia | Licensed | ([Who, 2011](#_heading=h.3j2qqm3)) |
|  | TBE-Moscow vaccine | Inactivated whole-virus | CFSC/Russia | Licensed | ([Ruzek et al., 2019](#_heading=h.35nkun2)) |
|  | Tick-E-Vac | Inactivated whole-virus | CFSC/Russia | Licensed | ([Who, 2011](#_heading=h.3j2qqm3)) |
|  | Enervac | Inactivated whole-virus | CFSC/Russia | Phase I, II | ([Vorovitch et al., 2020](#_heading=h.z337ya)) |
|  | SenTaiBao | Inactivated whole-virus | CDIBP /China | Licensed | ([Yoshii et al., 2017](#_heading=h.2xcytpi)) |
|  | LGT/DEN4 | RLA | NIAID | Phase I | ([Wright et al., 2008](#_heading=h.4i7ojhp)) |
| KFDV | KFD Vaccine | Inactivated whole-virus | Haffkine Institute/ Mumbai | Licensed | ([Kasabi et al., 2013](#_heading=h.1t3h5sf)) |

**Abbreviations: (YFV):** Yellow Fever Virus; **(DENV)**: Dengue virus; **(JEV):** Japanese Encephalitis Virus; **(WNV)** West Nile Virus; **(ZIKV)**: Zika virus; **(TBEV)**: Tick-Borne Encephalitis Virus; (**KFDV)**: Kyasanur Forest Disease Virus**; (RLA):** Recombinant Live-Attenuated, **(NIAID)** National Institute of Allergy and Infectious Diseases; **(OHSU)** Oregon Health & Science University, **(GSK)** GlaxoSmithKline; **(WRAIR)** Walter Reed Army Institute of Research; **(BIKEN)** Research Foundation for Microbial Diseases of Osaka University; **(CFSC)** Chumakov Federal Scientific Center; **(CDIBP)** Chengdu Institute of Biological Products. **NCT#**: Clinical trial code in www.clinicaltrials.gov

**REFERENCES**

Barrett, P.N., Terpening, S.J., Snow, D., Cobb, R.R., and Kistner, O. (2017). Vero cell technology for rapid development of inactivated whole virus vaccines for emerging viral diseases. *Expert Rev Vaccines* 16(9), 883-894. doi: 10.1080/14760584.2017.1357471.

Beckett, C.G., Tjaden, J., Burgess, T., Danko, J.R., Tamminga, C., Simmons, M., et al. (2011). Evaluation of a prototype dengue-1 DNA vaccine in a Phase 1 clinical trial. *Vaccine* 29(5), 960-968. doi: 10.1016/j.vaccine.2010.11.050 S0264-410X(10)01684-1 [pii].

Bista, M.B., Banerjee, M.K., Shin, S.H., Tandan, J.B., Kim, M.H., Sohn, Y.M., et al. (2001). Efficacy of single-dose SA 14-14-2 vaccine against Japanese encephalitis: a case control study. *Lancet* 358(9284), 791-795. doi: S0140673601059670 [pii] 10.1016/s0140-6736(01)05967-0.

Chin, R., and Torresi, J. (2013). Japanese B Encephalitis: An Overview of the Disease and Use of Chimerivax-JE as a Preventative Vaccine. *Infect Dis Ther* 2(2), 145-158. doi: 10.1007/s40121-013-0018-2.

Dayan, G.H., Bevilacqua, J., Coleman, D., Buldo, A., and Risi, G. (2012). Phase II, dose ranging study of the safety and immunogenicity of single dose West Nile vaccine in healthy adults >/= 50 years of age. *Vaccine* 30(47), 6656-6664. doi: 10.1016/j.vaccine.2012.08.063 S0264-410X(12)01281-9 [pii].

Demicheli, V., Debalini, M.G., and Rivetti, A. (2009). Vaccines for preventing tick-borne encephalitis. *Cochrane Database Syst Rev* (1), CD000977. doi: 10.1002/14651858.CD000977.pub2.

Durbin, A.P., Wright, P.F., Cox, A., Kagucia, W., Elwood, D., Henderson, S., et al. (2013). The live attenuated chimeric vaccine rWN/DEN4Delta30 is well-tolerated and immunogenic in healthy flavivirus-naive adult volunteers. *Vaccine* 31(48), 5772-5777. doi: 10.1016/j.vaccine.2013.07.064 S0264-410X(13)01050-5 [pii].

Kasabi, G.S., Murhekar, M.V., Sandhya, V.K., Raghunandan, R., Kiran, S.K., Channabasappa, G.H., et al. (2013). Coverage and effectiveness of Kyasanur forest disease (KFD) vaccine in Karnataka, South India, 2005-10. *PLoS Negl Trop Dis* 7(1), e2025. doi: 10.1371/journal.pntd.0002025 PNTD-D-12-00921 [pii].

Kikukawa, A., Gomi, Y., Akechi, M., Onishi, T., Manabe, S., Namazue, J., et al. (2012). Superior immunogenicity of a freeze-dried, cell culture-derived Japanese encephalitis vaccine (inactivated). *Vaccine* 30(13), 2329-2335. doi: 10.1016/j.vaccine.2012.01.054 S0264-410X(12)00092-8 [pii].

Kirkpatrick, B.D., Whitehead, S.S., Pierce, K.K., Tibery, C.M., Grier, P.L., Hynes, N.A., et al. (2016). The live attenuated dengue vaccine TV003 elicits complete protection against dengue in a human challenge model. *Sci Transl Med* 8(330), 330ra336. doi: 10.1126/scitranslmed.aaf1517 8/330/330ra36 [pii].

Ledgerwood, J.E., Pierson, T.C., Hubka, S.A., Desai, N., Rucker, S., Gordon, I.J., et al. (2011). A West Nile virus DNA vaccine utilizing a modified promoter induces neutralizing antibody in younger and older healthy adults in a phase I clinical trial. *J Infect Dis* 203(10), 1396-1404. doi: 10.1093/infdis/jir054 jir054 [pii].

Manoff, S.B., George, S.L., Bett, A.J., Yelmene, M.L., Dhanasekaran, G., Eggemeyer, L., et al. (2015). Preclinical and clinical development of a dengue recombinant subunit vaccine. *Vaccine* 33(50), 7126-7134. doi: 10.1016/j.vaccine.2015.09.101 S0264-410X(15)01391-2 [pii].

Monath, T.P., Fowler, E., Johnson, C.T., Balser, J., Morin, M.J., Sisti, M., et al. (2011). An inactivated cell-culture vaccine against yellow fever. *N Engl J Med* 364(14), 1326-1333. doi: 10.1056/NEJMoa1009303.

Rupp, R., Luckasen, G.J., Kirstein, J.L., Osorio, J.E., Santangelo, J.D., Raanan, M., et al. (2015). Safety and immunogenicity of different doses and schedules of a live attenuated tetravalent dengue vaccine (TDV) in healthy adults: A Phase 1b randomized study. *Vaccine* 33(46), 6351-6359. doi: 10.1016/j.vaccine.2015.09.008 S0264-410X(15)01251-7 [pii].

Ruzek, D., Avsic Zupanc, T., Borde, J., Chrdle, A., Eyer, L., Karganova, G., et al. (2019). Tick-borne encephalitis in Europe and Russia: Review of pathogenesis, clinical features, therapy, and vaccines. *Antiviral Res* 164, 23-51. doi: S0166-3542(18)30447-9 [pii] 10.1016/j.antiviral.2019.01.014.

Schuller, E., Klade, C.S., Wolfl, G., Kaltenbock, A., Dewasthaly, S., and Tauber, E. (2009). Comparison of a single, high-dose vaccination regimen to the standard regimen for the investigational Japanese encephalitis vaccine, IC51: a randomized, observer-blind, controlled Phase 3 study. *Vaccine* 27(15), 2188-2193. doi: 10.1016/j.vaccine.2008.12.062 S0264-410X(09)00135-2 [pii].

Sridhar, S., Luedtke, A., Langevin, E., Zhu, M., Bonaparte, M., Machabert, T., et al. (2018). Effect of Dengue Serostatus on Dengue Vaccine Safety and Efficacy. *N Engl J Med* 379(4), 327-340. doi: 10.1056/NEJMoa1800820.

Thomas, S.J., and Rothman, A.L. (2015). Trials and tribulations on the path to developing a dengue vaccine. *Vaccine* 33 Suppl 4, D24-31. doi: 10.1016/j.vaccine.2015.05.095 S0264-410X(15)00779-3 [pii].

Vorovitch, M.F., Grishina, K.G., Volok, V.P., Chernokhaeva, L.L., Grishin, K.V., Karganova, G.G., et al. (2020). Evervac: phase I/II study of immunogenicity and safety of a new adjuvant-free TBE vaccine cultivated in Vero cell culture. *Hum Vaccin Immunother* 16(9), 2123-2130. doi: 10.1080/21645515.2020.1757990.

Who, P. (2011). Vaccines against tick-borne encephalitis: WHO position paper--recommendations. *Vaccine* 29(48), 8769-8770. doi: 10.1016/j.vaccine.2011.07.024 S0264-410X(11)01045-0 [pii].

World Health Organization (2013). Vaccines and vaccination against yellow fever. WHO position paper -- June 2013. *Wkly Epidemiol Rec* 88(27), 269-283.

Wright, P.F., Ankrah, S., Henderson, S.E., Durbin, A.P., Speicher, J., Whitehead, S.S., et al. (2008). Evaluation of the Langat/dengue 4 chimeric virus as a live attenuated tick-borne encephalitis vaccine for safety and immunogenicity in healthy adult volunteers. *Vaccine* 26(7), 882-890. doi: 10.1016/j.vaccine.2007.12.015 S0264-410X(07)01479-X [pii].

Yoshii, K., Song, J.Y., Park, S.B., Yang, J., and Schmitt, H.J. (2017). Tick-borne encephalitis in Japan, Republic of Korea and China. *Emerg Microbes Infect* 6(9), e82. doi: 10.1038/emi.2017.69 emi201769 [pii].
