# Supplementary material for: Mucosal Vaccination: A Promising Alternative Against Flaviviruses
Source: Front Cell Infect Microbiol. 2022 Jun 15;12:887729. doi: 10.3389/fcimb.2022.887729 (PMC9241634; doi:10.3389/fcimb.2022.887729)
Supplement: Supplementary file 2 [file Table_2.docx]

Supplementary Table: Flavivirus mucosal vaccine candidates

| **TYPE OF VACCINE OR VECTOR** | **ANTIGEN AND ADJUVANT (IF APPLICABLE)** | **ANIMAL MODEL** | **MUCOSAL PATHWAY** | **ELICITED INMUNE RESPONSE** | **REFERENCE** |
| --- | --- | --- | --- | --- | --- |
| **Dengue virus (DENV)** | | | | | |
| Subunit | Domain III (DIII) of E protein from the four DENV serotypes and the capsid protein. Oligodeoxynucleotide 39M as an adjuvant. | Mice | Intranasal (IN) | Both intraperitoneal and IN routes elicited neutralizing antibodies. The mucosal route favored DENV-specific cell-mediated immunity. | ([Lazo Vazquez et al., 2017](#_heading=h.17dp8vu)) |
| Subunit (fusion protein) | Consensus DIII E protein from DENV was linked to cholera toxin B subunit (CTB) (adjuvant). This fusion protein was expressed in transgenic rice calli (*Oryza sativa* L). | BALB/c mice | Oral | Systemic and mucosal antibody responses, as well as cellular immune responses were elicited. | ([Kim et al., 2016](#_heading=h.26in1rg)) |
| Subunit (fusion protein) | NS3 region aa 296–618 from DENV2 was linked to peptide Co1 (M-cell targeting). This fusion protein was expressed in *Escherichia coli.* | BALB/c mice | Oral | Cellular protective immune response was demonstrated. | ([Kim et al., 2018](#_heading=h.lnxbz9)) |
| Subunit (fusion protein) | EDIII from all four DENV serotypes was linked to peptide Co1 (M-cell targeting). This fusion protein was expressed in *Saccharomyces cerevisiae.* | BALB/c mice | Oral | EDIII-Co1 fusion protein bound to the C5aR on the M cell surface, a preliminary requisite to induce immune response through oral mucosa vaccination. | ([Nguyen et al., 2015](#_heading=h.35nkun2)) |
| Subunit (fusion protein) | EDIII from all four DENV serotypes was linked to peptide Co1 (M-cell targeting). This fusion protein was expressed in transgenic rice calli. | BALB/c mice | Oral | Binding of ligand EDIII-Co1 chimeric protein to M cells on Peyer’s patches | ([Kim et al., 2013b](#_heading=h.1ksv4uv)) |
| Subunit (fusion protein) | EDIII of DENV2 was linked to OmpH of *Yersinia enterocolitica* (M-cell targeting). No expression system was declared. | BALB/c mice | Oral | Priming of the humoral and cellular responses in either systemic or mucosal compartments. Antibodies neutralized DENV, Th2 cytokine-secreting cell-mediated immunity. Responses were not tolerogenic. | ([Kim et al., 2013a](#_heading=h.44sinio)) |
| Nanoparticles (NPs) of chitosan and trimethyl chitosan | DIII of DENV3 E protein. | None | IN | *In vitro*, local innate antiviral immune response. | ([Nantachit et al., 2016](#_heading=h.1y810tw)) |
| NPs of oleic acid emulsion stabilized by chitosan | Recombinant tetravalent DENV antigen. | Mice | IN | Humoral and cellular antigen-specific immune response and mucosal immune response. | ([Vemireddy et al., 2018](#_heading=h.4i7ojhp)) |
| NPs of polycaprolactone | Recombinant tetravalent DENV antigen. | BALB/c mice | IN | Humoral and cellular antigen-specific immune response. | ([Vemireddy et al., 2019](#_heading=h.2xcytpi)) |
| Bacterial ghost from *Salmonella* *enterica* serovar Typhimurium | Envelope protein  domain III (EDIII) of all four DENV serotypes. | BALB/c mice | Oral | Humoral and specific cell-mediated immunity against EDIII of DENV. | ([Kim et al., 2020](#_heading=h.qsh70q)) |
| *S. enterica* serovar Typhimurium SL3261 | E protein of DENV4 | BALB/c mice | Oral | Specific antibody immune response. | ([Cohen et al., 1990](#_heading=h.2p2csry)) |
| *S. enterica* serovar Typhimurium SL3261 | NS1 protein of DENV2, Amphotericin B (AmB) as adjuvant. | BALB/c mice | Oral | IgG antibody immune response and protective efficacy. | ([Liu et al., 2006](#_heading=h.147n2zr)) |
| *S. enterica* serovar Typhimurium SL3261 | 298–306-amino acid of NS3 protein, CTL epitope of the DENV2. | BALB/c mice | Oral | CTL immune response. | ([Luria-Perez et al., 2007](#_heading=h.3o7alnk)) |
| *Lactococcus lactis* | EDIII of DENV2. | C57BL/6 mice | Oral and IN | Systemic neutralizing IgG antibodies. | ([Sim et al., 2008](#_heading=h.23ckvvd)) |
| *S. cerevisiae* | Consensus DIII of DENV2 E protein. | BALB/c mice | Oral | Systemic IgG humoral immune response, and mucosal IgA immune response. | ([Bal et al., 2018a](#_heading=h.ihv636)) |
| *S. cerevisiae* | Consensus DIII of DENV2 E protein. | BALB/c mice | Oral | Systemic IgG humoral immune response, and mucosal IgA immune response. | ([Bal et al., 2018b](#_heading=h.32hioqz)) |
| **Japanese encephalitis virus (JEV)** | | | | | |
| Inactivated | Mouse brain-derived formalin from whole virions of JEV. *Bordetella pertussis*, cholera toxin and/or pertussis toxin as adjuvants. | Mice | IN or oral | Inactivated vaccine plus bacterial adjuvants intranasally administered was the best combination. It showed antibody response comparable with parenteral immunization but with better cellular responses. | ([Harakuni et al., 2009](#_heading=h.3znysh7)) |
| Subunit | E protein of JEV expressed in transgenic rice and in *E.coli.* | BALB/c mice | Oral | Systemic and mucosal antibody responses. | ([Wang et al., 2009](#_heading=h.4d34og8)) |
| NPs of chitosan or chitosan maleimide | Live attenuated Japanese encephalitis chimeric virus vaccine (JE-CV). | C57/BL6 mice | IN | Seroprotection, higher sIgA levels, improved mucosal immune response. | ([Dumkliang et al., 2021](#_heading=h.1ci93xb)) |
| Liposomes | NS1 protein. Amphotericin B (AmB) as adjuvant. | Mice | Oral | NS1-specific IgG antibody response and protection against lethal JEV challenges. | ([Lin et al., 2010](#_heading=h.3whwml4)) |
| Replication-defective recombinant human adenovirus type 5 (rAd5) | PrM and E proteins of JEV. | BALB/c mice | Oral | Low titles of anti JEV and little JEV neutralizing activity. | ([Appaiahgari et al., 2006](#_heading=h.3as4poj)) |
| rAd5 | JEV E epitopes  (six amino acid residues 60–68, 327–333, 337–345, 373–399, 397–403 and 436–445 in E protein). | BALB/c mice | Oral | Humoral response with IgG anti JEV, JEV neutralizing activity, JEV virus Th1 immune response. | ([Li et al., 2008](#_heading=h.1pxezwc)) |
| **West Nile virus (WNV)** | | | | | |
| Subunit | E protein of WNV expressed in *Drosophila* cells. | Chickens (*Gallus gallus*) | Oral | Intramuscular vaccination yielded better results than oral route. | ([Fassbinder-Orth et al., 2009](#_heading=h.2s8eyo1)) |
| Subunit | DIII of E protein of WNV (no expression system was declared). Mast cell-activating compounds as adjuvants. | BALB/c mice | IN | Induction of specific and protective immune response when the immunogen was adjuvanted to mast cell-activating compounds. | ([Johnson-Weaver et al., 2021](#_heading=h.3rdcrjn)) |
| Subunit (fusion protein) | DIII from WNV linked to non-toxic cholera toxin CTA2/B domains (adjuvant). This fusion protein was expressed in *E. coli*. | BALB/c mice | IN | Fusion protein induced systemic and mucosal antibody responses, and increased IgG2a/IgG1 ratio. Antibodies can activate complement *in vitro.* DIII antigen was also effective in stimulating significant systemic IgG responses from increased dosage. | ([Tinker et al., 2014](#_heading=h.3j2qqm3)) |
| Microcapsules of alginate and spermidine | DNA vaccine encoding prM and E glycoproteins from WNV. | Fish crows (*Corvus ossigrafus*) | Oral | Oral administration did not elicit neutralizing antibodies, although it partially protected the animals after challenge. | ([Turell et al., 2003](#_heading=h.2bn6wsx)) |
| Vesicular stomatitis virus | E protein of WNV. | BALB/c mice | IN | Robust cellular immune response and strong neutralizing antibody response protected against lethal WNV challenge. | ([Iyer et al., 2009](#_heading=h.1hmsyys)) |
| Newcastle Disease virus | PrM and E proteins of WNV. | Chickens, ducks, and geese | IN and oral | Significant levels of WNV-specific IgG in chicken, duck, and geese models. | ([Wang et al., 2016](#_heading=h.41mghml)) |
| **Zika virus (ZIKV)** | | | | | |
| Attenuated | ZIKV PRVABC59 mutant strain lacking a glycosylation site in the E protein. | *Ifnar1^-/-^* mice of congenic C57BL/6 genetic background | Rectal | Rectal ZIKV inoculation led to subclinical and non-neurological disease outcomes. Protective immunity was reached in most of the immunized mice. | ([Martinez et al., 2020](#_heading=h.2et92p0)) |
| Subunit (fusion protein) | Three epitopes from ZIKV E protein were linked to B subunit of enterotoxigenic *E. coli*, a heat-labile toxin (LTB)(adjuvant). This fusion protein was expressed in marine microalgae *Schizochytrium* sp. | BALB/c mice | Oral | Fusion protein expressed in microalgae was immunogenic and induced systemic or mucosal responses when oral route was used. | ([Marquez-Escobar et al., 2018](#_heading=h.2jxsxqh)) |
| Subunit (fusion protein) | EDIII from ZIKV was linked to formyl peptide receptor-like 1 inhibitory protein (FLIPr)(FcγR antagonist). This fusion protein was expressed in *E. coli*. | AG129 mice (immunocompromised mice lacking receptor for IFN α/β/γ) | IN | Neutralizing systemic and mucosal antibody responses. Protective immunity was elicited. | ([Hsieh et al., 2021](#_heading=h.z337ya)) |
| rAd5 | PrM and E protein of ZIKV. | C57BL/6J mice | IN | Humoral and cellular immune response and CD8+ T cells specific for a dominant ZIKV T cell epitope, protection against a ZIKV challenge. | ([Steffen et al., 2020](#_heading=h.49x2ik5)) |
| **Thick-borne encephalitis virus (TBEV)** | | | | | |
| Recombinant TK variant of WR vaccinia strain | Structural and nonstructural TBEV  Proteins. | BALB/c mice | IN | Mucosal and humoral immune response against TBE and protection against TBE challenge. | ([Ryzhikov et al., 1998](#_heading=h.vx1227)) |
| NPs containing peptides and inactivated virus | Antigenic peptide 89–119 and inactivated TBEV strain Sofjin. | BALB/c mice | IN | TBEV-specific IgA antibodies; full protection against systemic challenge was afforded with inactivated vaccine; a lower protection was achieved by the peptide vaccine. | ([Goncharova et al., 2006](#_heading=h.3fwokq0)) |
| Virus-like microparticles  Liposomes  Attenuated Salmonella  Recombinant vaccinia | E protein of the TBEV  E protein of the TBEV  E protein of the TBEV  TBEV proteins C, prM, E, NS1, NS2a, NS2b, and NS3 | BALB/c mice | IN  IN  IN  IN | Protein E detected in transfected cells  Protein E detected in transfected cells  Protein E detected in transfected cells  TBEV Th1 immune response, protection against lethal TBEV challenge | ([Goncharova et al., 2002](#_heading=h.1v1yuxt)) |
| **Duck Tembusu Virus (DTMUV)** | | | | | |
| *S. enterica* serovar Typhimurium SL 7207 | C protein of DTMUV | Ducks | Oral | Discrete anti-C, neutralizing IgY antibodies. Higher survival rates during intravenous challenge. | (Huang et al., 2018) |
| *S. enterica* serovar Typhimurium SL 7207 | prM and E protein of DTMUV | Ducks | Oral | Discrete anti-E, neutralizing IgY antibodies. Higher survival rates during intravenous challenge. | (Huang et al., 2018) |
| **Louping ill virus (LIV)** | | | | | |
| Semliki forest virus | prME and  NS1 proteins of LIV. | BALB/c mice | IN | Cellular and humoral immune response against LIV, protection against  LIV fully virulent strain LI/31, and lower virulent LI/I challenge. | ([Fleeton et al., 1999](#_heading=h.2grqrue)) |
